# From Benign Parasite to Emerging Pathogen: A Review of *Theileria orientalis* in Ruminants

**DOI:** 10.3390/ani16152348

**Published:** 2026-08-01

**Authors:** Maria Felício, Sara Tudela Zúquete, Inês L. S. Delgado, Esmeralda Inácio, Sofia Nolasco, Pedro Caetano, Afonso P. Basto, Ludovina Padre, Alexandre Leitão

**Affiliations:** 1 MED Mediterranean Institute for Agriculture, Environment and Development & CHANGE Global Change and Sustainability Institute, Universidade de Évora, Largo dos Colegiais 2, 7004-516 Évora, Portugal; maria.felicio@live.com.pt (M.F.); siortz@uevora.pt (S.T.Z.); pcaetano@uevora.pt (P.C.); lpadre@uevora.pt (L.P.); 2 IIFA—Instituto de Investigação e formação Avançada, Universidade de Évora, Palácio do Vimioso, Largo Marquês de Marialva, Apartado 94, 7002-554 Évora, Portugal; 3 CIISA—Centre for Interdisciplinary Research in Animal Health, Faculty of Veterinary Medicine, University of Lisbon, 1300-477 Lisbon, Portugal; esmeralda.inacio@fmv.ulisboa.pt (E.I.); sofia.nolasco@essl.ipl.pt (S.N.); abasto@fmv.ulisboa.pt (A.P.B.); 4 AL4AnimalS—Associate Laboratory for Animal and Veterinary Sciences, 1300-477 Lisbon, Portugal; 5 FMV—Faculdade de Medicina Veterinária, Universidade Lusófona—Centro Universitário de Lisboa, Campo Grande, N.° 376, 1749-024 Lisboa, Portugal; ines.delgado@ulusofona.pt; 6 I-MVET (Investigação em Medicina Veterinária), Faculdade de Medicina Veterinária, Universidade Lusófona—Centro Universitário de Lisboa, Campo Grande, N.° 376, 1749-024 Lisboa, Portugal; 7 Centro de Ciência Animal e Veterinária (CECAV), Faculdade de Medicina Veterinária, Universidade Lusófona—Centro Universitário de Lisboa, 1749-024 Lisboa, Portugal; 8 Faculdade de Medicina Veterinária, Universidade José Eduardo dos Santos, Huambo, P.O. Box 2458, Angola; 9 Escola Superior de Saúde de Lisboa, Instituto Politécnico de Lisboa, 1990-096 Lisboa, Portugal; 10 Departamento de Medicina Veterinária, Escola de Ciências e Tecnologias, Universidade de Évora, 7002-554 Évora, Portugal

**Keywords:** *Theileria orientalis*, oriental theileriosis, bovine, tick-borne diseases, *Haemaphysalis longicornis*, major piroplasm surface protein, MPSP, anaemia

## Abstract

Once regarded as a benign, clinically overlooked organism, *Theileria orientalis* has undergone a paradigm shift in its perceived status and is now recognised as a tick-borne haemoparasite of clinical and economic importance. Severe outbreaks characterised by haemolytic anaemia, reproductive losses, and mortality, primarily associated with the Ikeda and Chitose genotypes, have been increasingly reported in Australasia, Asia, and North America. In Europe and parts of Africa, however, the epidemiological status of *T. orientalis* remains poorly defined, despite increasing molecular evidence of circulation and reports of predominantly subclinical infections. The parasite is transmitted transstadially in ixodid ticks, with *Haemaphysalis longicornis* as the principal confirmed biological vector, although vector competence remains unverified for most suspected tick species. This narrative review was based on a comprehensive PubMed search from database inception up to the date of initial manuscript submission, supplemented by reference-list screening and targeted searches. This review synthesises current knowledge on taxonomy, life cycle, transmission, genetic diversity based on the major piroplasm surface protein gene, global epidemiology, pathogenesis, clinical manifestations, co-infections, production losses, diagnostics, and control strategies. Key gaps include limited understanding of the mechanisms driving erythrocyte destruction, insufficient experimental confirmation of vector competence, absence of licensed vaccines, and incomplete epidemiological data in several regions. Future research priorities are discussed in the context of climate change and the expanding geographic distribution of tick vectors.

## Simple Summary

*Theileria orientalis* is a tick-transmitted parasite that infects cattle worldwide and was long considered harmless. However, in recent decades, severe outbreaks causing anaemia, reproductive losses, and deaths have been reported in several countries, drawing increasing attention from the veterinary and scientific communities. Disease severity depends largely on the parasite genetic type, with the Ikeda and Chitose genotypes being the most dangerous. Diagnosis can be challenging, particularly in animals that carry the parasite without showing clinical signs. Treatment options remain limited, and no vaccine is currently available. Based on a comprehensive search of the available scientific literature, this narrative review brings together current knowledge on how this parasite is classified, how it spreads, how it causes disease and how infections can be managed. It also highlights the areas where research is still lacking, including a better understanding of which tick species can transmit the parasite and the development of effective vaccines. As tick distributions continue to expand due to climate change, understanding and controlling this infection are becoming increasingly important.

## 1. Introduction

Tick-borne diseases represent a threat to livestock health and welfare worldwide. Among these, bovine theileriosis, caused by protozoan parasites of the genus *Theileria* (Apicomplexa: Piroplasmorida Wenyon 1926), is one of the most economically important diseases affecting cattle [1,2]. While the transforming species *Theileria parva* and *Theileria annulata* have historically attracted most research attention [3], a growing body of evidence has brought renewed focus to a group of non-transforming species collectively referred to as the *Theileria sergenti/buffeli/orientalis* complex [4,5]. The taxonomic classification of this group has long been debated, with different names assigned based on geographic origin—*Theileria sergenti* in East Asia, *T. buffeli* in Australia, and *T. orientalis* in Europe—resulting in a complex nomenclature [5,6,7]. Although *T. orientalis* is now widely used as a collective designation for these parasites, their formal taxonomic status remains unresolved [2,8,9,10,11]. The WOAH Terrestrial Manual considers the *T. orientalis/buffeli* group to comprise two species, *T. orientalis* and *T. buffeli*, whereas much of the recent molecular and epidemiological literature describes Buffeli as a genotype within the *T. orientalis* complex [10,11,12,13]. For consistency with the predominant usage in the recent literature, *T. orientalis* is used throughout this review as the principal current designation. The names *T. buffeli* and *T. sergenti* are retained only when discussing historical nomenclature or when referring to the terminology used in the original publications.

Traditionally regarded as a benign or low-virulence parasite, *T. orientalis* has undergone a substantial shift in perceived clinical importance, evolving from an organism historically associated primarily with subclinical or mild infections to an emerging agent of bovine theileriosis [14]. This change in perception has been particularly evident in Australia and New Zealand, where outbreaks of disease have been linked to the Ikeda genotype and characterised by haemolytic anaemia, jaundice, weakness, reduced production, abortion and, in severe cases, mortality [15,16]. Reports from Asia and North America have since confirmed that clinically relevant *T. orientalis* infections are not restricted to Australasia [17,18,19,20,21], while recent European studies indicate circulation but still provide limited evidence regarding genotype distribution, vector associations and clinical relevance [22,23,24]. At least twelve genotypes have been identified based on the major piroplasm surface protein (MPSP) gene [25,26], of which the Ikeda (type 2) and Chitose (type 1) genotypes are most frequently associated with disease [10], while the widely distributed Buffeli genotype (type 3) is generally associated with clinically mild infections [14].

Despite growing recognition of its clinical and economic significance, important knowledge gaps remain. The vector competence of many ixodid tick species has not been experimentally confirmed [10], the pathogenic mechanisms underlying erythrocyte destruction remain incompletely characterised [13] and no licensed vaccines or consistently effective treatments are currently available [27]. Furthermore, although molecular evidence of *T. orientalis* circulation has been reported in several European countries, its true prevalence, genotype distribution, vector association, and clinical relevance in Europe remain insufficiently characterised [22,23,24]. This review aims to provide a comprehensive and updated synthesis of the current knowledge on *T. orientalis*, covering its taxonomy, morphology, life cycle, transmission, genetic diversity, epidemiology, pathogenesis, diagnosis, and control strategies, with the goal of identifying research priorities to guide the management of this emerging parasitic disease.

## 2. Methods—Literature Search Strategy

This narrative review was based on a comprehensive PubMed search from database inception up to the date of initial manuscript submission. No publication-date restriction was applied. Current and historical nomenclature was used, including “*Theileria orientalis*”, “*Theileria buffeli*”, “*Theileria sergenti*”, “oriental theileriosis” and “benign theileriosis”. Publications were considered when they addressed taxonomy, morphology, life cycle, transmission, genetic diversity, epidemiology, pathogenesis, clinical manifestations, productive impact, diagnosis, treatment or control of *T. orientalis*. Additional publications were identified by screening the reference lists of relevant articles and reviews, conducting targeted searches and consulting authoritative institutional sources.

The evidence was interpreted considering the design and limitations of each study. Experimental studies were given greater weight when assessing causal mechanisms and vector competence, whereas observational studies, molecular surveys and case reports were interpreted more cautiously. As this was a narrative review, no formal risk-of-bias assessment or quantitative evidence-grading system was applied.

**Figure 1:**
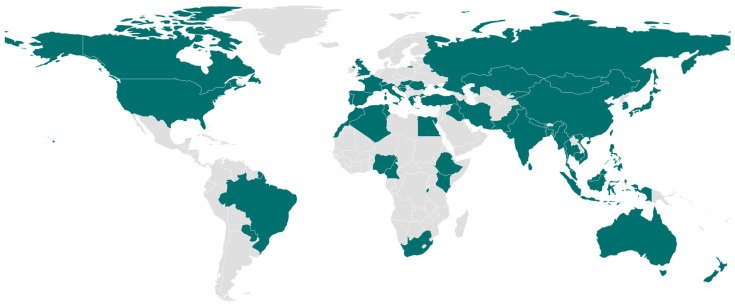
Global distribution of reported *Theileria orientalis* occurrence. Countries shown on the map had at least one eligible published record, although the type and strength of evidence varied among countries. Country-level evidence, diagnostic methods, reported genotypes, clinical relevance and vector associations are detailed in Supplementary Table S1.

Country-level records used to construct Figure 1 were extracted into a predefined table. For each country, information was recorded on the study area, host or sample type, type of evidence, diagnostic method, sample size and reported prevalence, genotype, clinical relevance, vector evidence and supporting reference. Records were checked against the original publications and are presented in Supplementary Table S1.

A country was considered to have reported occurrence when at least one eligible publication documented *Theileria orientalis* through molecular, serological, microscopic or clinical evidence. Because these approaches provide different levels of taxonomic and epidemiological certainty, the type and strength of evidence were recorded separately in Supplementary Table S1.

## 3. Taxonomy, Historical Background and Morphology

### 3.1. Historical Nomenclature and Current Taxonomic Status

The nomenclature of the non-transforming bovine *Theileria* lineages associated with oriental theileriosis has been debated for decades [5,6]. Historical designations were based largely on geographic origin, host species, morphology, serological reactivity and transmission characteristics, but these names have not consistently corresponded to the genetic lineages recognised today. Consequently, *T. sergenti*, *T. buffeli* and *T. orientalis* have been used inconsistently and sometimes interchangeably in the literature [5,6,10]. In the present review, *T. orientalis* is used as the principal current designation, whereas *T. buffeli* and *T. sergenti* are retained only for historical context or when reproducing the terminology of the original studies. The historical nomenclature reflects descriptions made at different times and in different hosts and geographic regions. The earliest of these names was *T. buffeli*, described by Schein in 1908 in Asian water buffalo (*Bubalus bubalis*) in Indochina, now Vietnam. *Theileria sergenti* was subsequently described by Yakimoff and Dekhtereff in 1930 in cattle from eastern Siberia, followed by the description of *T. orientalis* in cattle from the same region in 1931 [5,9]. However, the name *T. sergenti* had already been used by Wenyon in 1926 for a parasite of sheep, making its subsequent application to the bovine parasite taxonomically problematic [5,7].

In Australia, Dodd reported a bovine piroplasm in 1910 that was identified at the time as *T. mutans*, a species previously described in South African cattle [28,29]. Subsequent studies showed that the Australian parasite was distinct from the African *T. mutans* and was more closely related to the lineages later referred to as *T. buffeli* or *T. orientalis* [6,9].

Comparative studies conducted during the 1980s initially suggested that bovine *Theileria* isolates from Japan, Australia, Great Britain, Iran, the United States and Korea belonged to a single species, for which the name *T. orientalis* was provisionally retained [5,30,31]. However, subsequent transmission and serological studies showed that the Japanese parasite historically referred to as *T. sergenti* could be distinguished from the Australian *T. buffeli* and British *T. orientalis*, whereas the latter two appeared more closely related [5,6,9,32]. Two-dimensional protein analysis showed that the profiles of *T. buffeli* and *T. orientalis* were nearly identical, apart from a small number of minor proteins, whereas the Japanese *T. sergenti* stocks differed by a characteristic group of proteins that included a major 33–34 kDa antigen [33]. Nucleotide-sequence analysis of the genes encoding the immunodominant p33 protein of *T. sergenti* and the corresponding p34 protein of *T. buffeli* further supported differentiation between these parasites [34]. Marked morphological similarity between the schizont and piroplasm stages of *T. buffeli* and *T. orientalis* was also reported, although morphology alone was insufficient to resolve their taxonomic status [35]. In addition, *T. sergenti* was considered an unacceptable name for the bovine parasite because it had previously been used for an ovine parasite described as *Babesia sergenti* [5,7]. Taken together, these findings showed that the historical names could not simply be regarded as geographic synonyms and highlighted the heterogeneity within the group.

From the 1990s onwards, molecular analyses based on the major piroplasm surface protein (MPSP) and small-subunit ribosomal RNA genes confirmed the close relationship among parasites historically designated as *T. sergenti*, *T. buffeli* and *T. orientalis*, while also revealing considerable genetic heterogeneity within the group [4,5,36,37]. These findings led to the widespread use of the terms *T. sergenti/buffeli/orientalis* complex or *T. buffeli/orientalis* group, and much of the subsequent literature adopted *T. orientalis* as a collective designation, with the principal lineages described as genotypes [8,9,10,38]. Nevertheless, this convention did not formally resolve the taxonomic status of the lineages, and inconsistent nomenclature remains in use. A related non-transforming bovine parasite, *T. sinensis*, was subsequently described in China. Although phylogenetically related to other bovine *Theileria*, analyses of the internal transcribed spacer regions and the 5.8S rRNA gene placed *T. sinensis* in a distinct clade separate from *T. orientalis*, supporting its classification as a separate species rather than as another genotype of *T. orientalis* [39].

Whole-genome comparisons have provided stronger evidence of divergence among the principal lineages. Analysis of Australian Ikeda, Chitose and Buffeli isolates, together with the Japanese Ikeda reference genome, identified average nucleotide identity values comparable to those observed between some recognised *Theileria* species [11]. On this basis, the authors suggested that Ikeda and closely related genotypes could potentially be considered a species distinct from Chitose and Buffeli. However, the study did not constitute a formal taxonomic revision and was based on a limited number of representative genomes; it also identified putative recombination between Chitose and Buffeli and between Australian and Japanese Ikeda isolates, further complicating the definition of clear species boundaries [11]. Subsequent chromosome-level assemblies of the Chitose and Buffeli genotypes confirmed substantial genomic and structural differences, including chromosomal translocations, while continuing to classify these lineages as genotypes of *T. orientalis* [40]. Therefore, the available genomic evidence supports a reassessment of the current classification but does not yet establish a formally accepted species-level separation among Ikeda, Chitose and Buffeli [11,40].

### 3.2. Morphology

*Theileria orientalis* belongs to the genus *Theileria*, family *Theileriidae,* order Piroplasmida, class Aconoidasida and phylum Apicomplexa [8,41]. Parasites belonging to the phylum Apicomplexa are obligate intracellular parasites and propagate only within host cells. The genus *Theileria* is characterised by the presence of schizonts in lymphoid cells and piroplasms in the erythrocytes of the vertebrate host [2,8,9,42,43]. Microscopic detection of this haemoparasite is commonly performed using *Giemsa*-stained peripheral blood smears. Piroplasms typically measure up to 2.0 µm in length and 1.0 µm in width, while erythrocytes may exhibit spiculated (spiny) morphological changes during acute infection [44]. Schizonts in lymphoid cells are generally not observed on peripheral blood smears [42].

Intraerythrocytic piroplasms of *T. orientalis* are pleomorphic, most commonly appearing as bacilliform, ovoid, Y-shaped, tetrad, comma-shaped, and thick or thin rod-like forms with residual cytoplasm, as well as annular forms such as signet-ring and parachute-like forms [45,46]. At low parasitaemia, rod or comma forms tend to predominate, whereas later in infection the number of oval forms increases. Y-shaped and tetrad forms within erythrocytes are reported to exhibit multiple nuclei, suggesting multiplication by binary fission [45]. Piroplasms are sometimes associated with an intraerythrocytic “bar” or “veil”, formed in the cytoplasm of erythrocytes infected with piroplasms of several non-transforming *Theileria* species; this veil contains haemoglobin and is thought to be part of a detoxification system [44,45,47].

Morphological distinction on stained blood smears may be insufficient to reliably distinguish *T. orientalis* from *T. annulata*, as their intraerythrocytic merozoite forms can present overlapping features [1,48]. Differentiation from other haemoprotozoa and rickettsiae can also be difficult [10,44] and the low parasitaemia commonly observed in carrier animals further reduces the sensitivity of blood smear examination [1,25].

## 4. Life Cycle and Transmission

### 4.1. Life Cycle

The haemoprotozoan *T. orientalis* is an apicomplexan parasite with stages alternating between ticks and ruminant hosts such as cattle, water buffaloes, Cape buffaloes and yaks [2,49,50]. Its life cycle therefore includes a vertebrate as the intermediate host, in which asexual propagation by schizogony occurs, and a tick as the definitive host, in which sexual reproduction (gametogony) and asexual propagation by sporogony take place [8,43].

The vertebrate host becomes infested with ticks, which inoculate sporozoites during feeding; the sporozoites subsequently multiply and develop into schizonts within leucocytes of lymphoid organs [8,43]. These schizonts release uninucleate merozoites that infect erythrocytes, where further multiplication (merogony) occurs [43,51]. Erythrocyte invasion by merozoites occurs approximately 10 days after inoculation of the agent [42]. It has been suggested that *T. orientalis* utilises haemoglobin as a source of amino acids [13,52] during the intraerythrocyte phase, targeting host haemoglobin as a vital cytosolic nutrient source to support rapid proliferation. A potential example of host-protein interaction in *T. orientalis* is Tocp1, a piroplasm-expressed protein with sequence similarity to eukaryotic thiol proteases. Despite this similarity, Tocp1 does not show detectable proteolytic activity, probably because the catalytic cysteine residue is replaced by glycine. However, recombinant Tocp1 binds bovine haemoglobin in vitro, and several Tocp1-like genes are present in the parasite genome, suggesting a possible role in haemoglobin interaction during the intraerythrocytic stage [52].

When a new tick feeds on the vertebrate host, it ingests infected erythrocytes [43,51]. These erythrocytes are lysed in the intestinal lumen, releasing merozoites that differentiate into gametocytes, leading to sexual reproduction in the midgut (gametogony), with genetic recombination occurring at meiosis [53]. Following this division, the parasites differentiate into motile kinetes, which exit the tick’s intestinal cells, access the haemocoel and reach the salivary glands, where sporogony takes place. In this phase, the parasite develops into a multinucleate sporont that gives rise to multiple sporozoites, which are inoculated into the vertebrate host during tick feeding [51].

*T. orientalis* is transmitted transstadially, but not transovarially, in ticks [8,45]. Accordingly, recognised vectors are typically tick species with two- or three-host life cycles [9,49].

### 4.2. Confirmed and Suspected Tick Vectors

The primary vector associated with the biological transmission of *T. orientalis* is *Haemaphysalis longicornis* [54,55]. However, other ixodid species have been implicated in transmission, namely *Haemaphysalis bancrofti, Haemaphysalis humerosa, Haemaphysalis punctata* and *Rhipicephalus microplus* [10,54,56,57,58]. *Haemaphysalis longicornis* is native to East Asian countries, including Russia, China, Korea and Japan [59]. It can survive across a wide range of climatic conditions, and some populations are capable of parthenogenetic reproduction [54]. In addition, *H. longicornis* has been recorded on multiple host species, including horses, pigs, sheep, dogs and cats, although cattle are its primary host [60]. This ixodid tick has successfully invaded and established in several Asia-Pacific countries, including Australia and New Zealand. More recently, its presence has been confirmed in the USA [54] and it has also recently been recorded in Turkey [61].

*Haemaphysalis* spp. are the main biological vectors of *T. orientalis* in Australasia and Asia, with *H. longicornis* considered the principal vector in Australia, New Zealand and East Asia [49]. In the United States, invasive populations of *H. longicornis* have been experimentally confirmed as competent vectors of the Ikeda genotype [54], and *T. orientalis* Ikeda has more recently been detected in *H. longicornis* ticks collected from a cattle farm in Tennessee [62]. In Europe, *H. punctata* has been implicated as the most likely vector of *T. orientalis*, although this association remains less well characterised than that of *H. longicornis* in Australasia and the USA [58,63]. In Africa, vector associations for *T. orientalis* remain poorly defined, despite molecular evidence of parasite circulation [10,64,65,66].

The competence of some proposed vectors has not been established, as ticks were collected from cattle rather than from pasture, and no experimental infections with *T. orientalis* using those ticks have yet been demonstrated [10]. Consequently, there is a degree of inconsistency regarding agent detection versus confirmed transmission capacity among ixodids. Table 1 summarises selected species and their status with respect to *T. orientalis* presence or competence, according to various authors.

The number of infected tick acini appears to increase with bovine parasitaemia, probably reflecting the number of parasitised erythrocytes ingested during feeding [55]. Understanding transmission modes is essential for a comprehensive assessment of disease epidemiology and for the rational formulation of outbreak control measures [84].

The evidence supporting vector competence varies considerably among tick species. Controlled transmission experiments provide direct evidence for competence, whereas molecular detection of parasite DNA in field-collected ticks demonstrates exposure or carriage but not the ability to support parasite development and transmit infection to cattle. Epidemiological associations should therefore be interpreted as hypotheses requiring experimental confirmation.

### 4.3. Alternative Modes of Transmission

#### 4.3.1. Vertical Transmission

Vertical transplacental transmission of *T. orientalis* genotype Ikeda appears unlikely in chronically infected dairy cows and is therefore not considered a major factor in the epidemiology of Ikeda-type infection [85]. However, transplacental transmission of the parasite from cows to calves has been reported in approximately 5.5–13% of cases [54,86], suggesting that it may occur sporadically but is unlikely to maintain infection at the population level. Nevertheless, calves may develop high-level *T. orientalis* infections at 4–8 weeks of age, regardless of the presence of maternal antibodies after birth [87]. This subject remains controversial, as some authors report that vertically infected calves may take up to three months to test positive via PCR [86]. *T. orientalis* DNA has also been detected in aborted ovine and caprine material, although molecular detection alone does not establish causation [88,89].

#### 4.3.2. Iatrogenic and Mechanical Transmission

Reports of *T. orientalis* infection in settings where ixodid ticks are absent, rare or not detected at the time of sampling suggest that alternative transmission routes may contribute to parasite maintenance. Mechanical transfer of infected blood has been shown experimentally to establish persistent, PCR-detectable infection in recipient cattle, indicating that this route can contribute to dissemination. Nevertheless, because mechanical transmission bypasses the tick stage and does not consistently reproduce the severe disease associated with biological tick-borne transmission, passage through the tick vector has been proposed to be important for parasite fitness, genetic diversity and possibly virulence [21,90,91].

Successful infection with *T. orientalis*, detected by PCR, has been achieved by inoculating approximately 10^7^ to 10^8^ infected bovine erythrocytes, both intravenously and subcutaneously [84,91]; infection following intravenous inoculation with small volumes of blood can be detected by PCR for up to five months post-infection [84].

The onset of parasitaemia was delayed in animals infected intravenously compared with animals infected by ixodid ticks [54]. This may reflect the tick’s ability to inject protozoa together with immunomodulatory salivary proteins, amplification of parasite numbers within the vector, and/or additional multiplication in leucocytes (schizogony) before reaching erythrocytes [54]. Stabilised blood lacks these biological advantages and undergoes immunological degradation and destruction of infected cells [54]. Therefore, iatrogenic transmission depends on the volume and parasitaemia of the transferred blood and on the parasite’s ability to survive outside the host before inoculation into a susceptible animal [84].

Mechanical transmission is associated not only with contaminated needles but also with biting flies, mosquitoes and lice [49,84,90]. In these insects, regurgitation of part of a previous blood meal or passive transfer of blood on mouthparts are possible transmission modes [84]. Although confirmatory evidence of *T. orientalis* mechanical transmission is still limited, mechanical transfer of *Theileria* spp. has been reported, under certain conditions, by the horse fly *Tabanus trigeminus*, and of the mechanical transmission of *T. orientalis* by the sucking louse *Linognathus vituli*, in which mechanical transmission was suggested to occur by regurgitation [84]. A high frequency of *T. orientalis* detection has also been reported in *Haematopinus eurysternus*, but no transmission mechanisms were reported [90]. The agent has also been detected in flies, notably *Haematobia irritans* and *Stomoxys* spp. [90]. The detection of *T. orientalis* in *Culiseta annulata* mosquitoes in the United Kingdom and in the biting midges *Culicoides brevitarsis* and *Culicoides victoriae* in Australia has also been demonstrated [90,92]. However, for biting arthropods, transmission would depend on both donor parasitaemia and the cumulative volumes inoculated or transferred [84]. Because mechanical transmission results in direct transfer of the haploid stage from host to host, the sexual phase of the life cycle does not occur [84] and the life cycle is interrupted [2,9]. Consequently, genetic exchange has not been confirmed in experimental studies of mechanical transmission, although this phenomenon may play an important role in the persistence and spread of theileriosis. Extensive mechanical transmission may facilitate parasite dissemination by transferring infected blood-stage parasites between cattle and establishing persistent, PCR-detectable infection. However, in an experimental study mechanical inoculation produced infection without reproducing overt clinical disease, suggesting that this route may disseminate the parasite without fully maintaining the pathogenic phenotype observed after tick-borne transmission. The authors therefore proposed that biological transmission through ticks may be required to maintain parasite virulence [84].

Although the minimum infectious dose of *T. orientalis* is very low, the risk of mechanical spread caused by biting arthropods depends on several interacting factors. These include the prevalence of infection within the herd, the parasitaemia level of infected donor animals, the volume of blood transferred to a naïve host, and the parasite load in the arthropod’s previous blood meal [84]. Transmission is also influenced by the number of arthropods feeding within the same period, their ability to reach a susceptible recipient shortly after an interrupted blood meal, or, in the case of lice, their transfer between animals through direct contact [84]. Ultimately, successful mechanical transmission requires viable parasites to remain associated with the retained blood and to be inoculated in sufficient quantities to establish a detectable and potentially transmissible infection [84].

Approximately 10% of calves born to infected dams may become PCR-positive within the first three months of life, despite testing negative at birth [86]. This is consistent with the epitheliochorial placenta of ruminants, which separates maternal and foetal blood supplies and restricts prenatal transfer of macromolecules such as immunoglobulins [87]. Passive immune transfer therefore occurs mainly after birth through colostrum, which contains biologically active maternal components, including immunoglobulins, cytokines and viable leucocytes [93,94]. Although transplacental transmission of *T. orientalis* can occur, it appears to occur at a relatively low rate and is unlikely to be a direct cause of abortion; instead, abortion may be more closely associated with the maternal clinical state and high maternal parasitaemia [87].

Calves may acquire colostral antibodies against *T. orientalis*, but their protective role remains unclear. Maternal antibodies may provide only partial and transient protection against severe disease or mortality, as they do not appear to prevent infection, parasitaemia or anaemia; parasite loads often increase after antibody levels decline, around 5–8 weeks postpartum [49,87].

Overall, biological transmission by competent ixodid ticks remains the best-supported and epidemiologically most important route. Mechanical transfer can establish persistent infection experimentally, but its contribution under field conditions remains uncertain because the infectious blood volume, donor parasitaemia and frequency of interrupted feeding are rarely quantified. Transplacental transmission has been demonstrated at a low frequency in observational studies and is unlikely to maintain infection at the population level.

## 5. Genetic Diversity and Genotypes

It is estimated that the ancestor of extant piroplasmids originated around 57 million years ago [3]. Molecular studies indicate that transforming and non-transforming *Theileria* species evolved independently [3]. These studies have also provided useful insights into the epidemiology, diagnosis, taxonomy and phylogeny of benign *Theileria* species across various regions. These studies have primarily targeted the following genes: 18S ribosomal RNA (rRNA), internal transcribed spacers (ITS), major piroplasm surface protein (MPSP), the 23 kDa piroplasm membrane protein (p23), among other genetic markers [38,95,96,97]. Genetic variation among *Theileria* parasites is sometimes associated with host specificity, but the genetic diversity of *T. orientalis* is less extensively studied than that of *T. annulata* and *T. parva* [3]. Recent molecular studies in Bangladesh have revealed considerable genetic diversity and distinct population structures among circulating *T. orientalis* genotypes, highlighting the complexity of parasite populations in endemic Asian regions [98].

*T. orientalis* is classified on the basis of its morphology, vector specificity, pathogenicity and 18S small subunit ribosomal RNA or MPSP sequences [38].

Phylogenetic analysis of MPSP sequences has revealed 12 MPSP types (type 1/Chitose, type 2/Ikeda, type 3/Buffeli, types 4–8, N1, N2, N3 and N4) to date, some of which correlate with virulence [8,14,26,99,100]. The Chitose genotype can be further classified into Chitose A and Chitose B [99].

Types 1 and 2 (Chitose and Ikeda, respectively) are known to be more virulent and to cause severe health effects in animals, whereas the widely distributed type 3 (Buffeli) is mainly associated with benign/mild infections [10,14,15]. The Ikeda genotype is considered the most virulent [10,14,15], with recent studies indicating the possibility that this genotype represents a distinct species from the Chitose or Buffeli genotypes [8].

Evidence linking genotype to clinical outcome is strongest for the Ikeda genotype, because associations identified during field outbreaks are supported by controlled experimental infections demonstrating haematological deterioration. For Chitose and the less common types 4, 7 and N2, evidence is based predominantly on outbreak investigations, case reports or observational associations and is therefore less conclusive. Genotype alone is also unlikely to determine clinical outcome, as parasite burden, mixed-genotype infection, previous exposure, host susceptibility and physiological stress may modify disease expression. Consequently, the detection of a genotype associated with virulence should not be interpreted as evidence that all infected animals will develop clinical disease.

## 6. Epidemiology

### 6.1. Global and Regional Distribution

The cosmopolitan distribution of *T. orientalis* has been attributed to the global movement of cattle without consideration of infection status, and its occurrence depends primarily on the availability of a suitable vector [10,49]. It has been detected on several continents, including Asia, Africa, Oceania, the Americas and Europe; however, clinical outbreaks are predominantly reported in parts of Asia, Oceania and North America [1,22,54,64,101], although the type and strength of the available evidence vary markedly among regions. Among the reported genotypes, Ikeda is the most consistently associated with severe clinical disease and has been documented across Australasia, East and South Asia, and North America (Supplementary Table S1). Its distribution is therefore broader than the regions in which the largest and most extensively investigated outbreaks have occurred.

Australasia represents the most extensively characterised epidemiological setting [49]. In Australia and New Zealand, molecular surveillance has demonstrated the circulation of multiple genotypes, including Ikeda, Chitose and Buffeli [102,103,104], although clinical outbreaks and measurable production losses have been most consistently associated with Ikeda [103,105,106]. The availability of longitudinal studies, outbreak investigations and confirmed vector data contrasts with the more fragmentary evidence available from several other regions (Supplementary Table S1).

East and Southeast Asia show broad parasite circulation and substantial genetic diversity [21,71,107,108,109,110,111,112,113]. Buffeli, Ikeda, Chitose, types 5 and 7 have been reported across the region [71,107,108,112,113], together with historical MPSP allelic categories that are not always directly comparable with the current numbered genotype system [109,110]. While severe disease associated with Ikeda and other potentially pathogenic genotypes has been documented in China and parts of South Asia [21,99], many surveys in Japan and Southeast Asia primarily identified apparently healthy carriers [71,107,108,109,111,112,113] (Supplementary Table S1).

In South, Western and Central Asia, molecular studies confirm widespread occurrence but reveal marked variation in study design and clinical interpretation [50,99,114,115,116,117,118,119,120,121,122,123,124]. Buffeli and Chitose predominate in several surveys [116,118,119,120,122,123,124,125], whereas Ikeda and other genotypes have also been identified, particularly in Pakistan and India [99,122]. India provides some of the strongest regional evidence of severe and fatal disease [99], while evidence from Central Asia is based mainly on cross-sectional surveys of apparently healthy cattle [116,118,119] (Supplementary Table S1).

European evidence is geographically dispersed and methodologically heterogeneous [23,24,73,92,125,126,127,128,129,130,131,132,133,134]. Molecular detection in cattle has been reported in several countries [23,24,73,125,126,127,129,131,132,133,134], but genotype information is often unavailable [23,24,73,126,127,129,132,133,134], and most studies describe asymptomatic or subclinical infection [23,24,73,125,132]. Clinical disease has been reported occasionally, including in Hungary and Croatia [73,126], whereas evidence from some countries is limited to historical serology [128], arthropod blood meals [92] or parasite DNA detected in ticks [130]. Competent local vectors remain unconfirmed in most European settings [73,92,129,130,132,133] (Supplementary Table S1).

In Africa, the evidence remains comparatively fragmented [64,65,66,135,136,137,138,139,140,141,142,143,144]. Molecular detection in cattle has been reported in several northern and sub-Saharan countries [64,66,135,136,137,138,139,140,141,142,143,145], whereas other records derive from wildlife hosts [136], ticks [66] or historical outbreak investigations, such as the report from Burundi [65]. Genotype characterisation, clinical follow-up and experimental vector studies remain limited, restricting interpretation of the parasite’s epidemiological and economic relevance across the continent [64,65,66,136,137,138,139,140,141,142] (Supplementary Table S1).

In the Americas, the epidemiological picture differs markedly between regions [54,100,146,147,148,149,150,151]. In the USA, Ikeda has emerged as a clinically relevant genotype and transmission by invasive *H. longicornis* has been demonstrated [54,150]. Canadian evidence is more limited and includes individual cases [148,149], whereas South American records are based mainly on molecular studies in cattle or buffaloes, with limited information on clinical and productive consequences [100,146,147] (Supplementary Table S1).

Reported prevalence varied widely among countries and studies; however, direct numerical comparisons should be interpreted cautiously. The studies differed in host population, clinical status, geographic coverage, sampling strategy, diagnostic target and assay sensitivity. In addition, some records were based on vertebrate-host infection, whereas others reflected only serological exposure or parasite DNA in arthropods. Figure 1 presents the global distribution of reported *T. orientalis* occurrence based on the country-level evidence summarised in Supplementary Table S1 [21,23,24,50,54,64,65,66,73,92,97,99,100,102,103,104,107,108,109,110,111,112,113,114,115,116,117,118,119,120,121,122,123,124,125,126,127,128,129,130,131,132,133,134,135,136,137,138,139,140,141,142,143,144,146,147,148,149,150,151,152,153,154,155]. The supporting evidence, diagnostic approach, genotype information, clinical relevance and vector associations for each country are provided in the Supplementary Material.

### 6.2. Epidemiological Determinants and Enzootic Stability

Seasonal fluctuations play a key role in its epidemiology: in Holstein cattle grazing in the Republic of Korea, infection rates increased progressively from 11% in March to 60% in November, mirroring the seasonal peak in tick infestation [156,157]. Grazing in mountainous areas and extensive management systems have been closely associated with increased infection rates [156,158,159]. However, there are reports of outbreaks in housed animals, indicating that the disease is not exclusive to grazing systems [21]. The epidemiological impact of *T. orientalis* is strongly influenced by the level of enzootic stability within a given region. In areas of enzootic stability, most calves are exposed early in life and develop immunity during the period of passive protection conferred by maternal antibodies, resulting in a low incidence of disease in adults [49]. Conversely, in regions of enzootic instability, characterised by low tick infection rates and/or low tick infestation pressure, many animals reach adulthood without prior exposure to the parasite, rendering them highly susceptible to severe clinical episodes upon first infection [49]. In Australia and New Zealand, *T. orientalis* is regarded as an established and economically important cattle parasite in affected regions, with outbreaks of oriental theileriosis causing substantial concern and production losses in both dairy and beef systems. In the United States, the emergence of Ikeda-type infection is increasingly recognised as a potential threat to the cattle industry [17,18,54,62,160]. In endemic regions, resident cattle may develop herd-level immunity following repeated exposure to *T. orientalis*. However, clinical outbreaks can occur when immunologically naïve cattle from non-endemic areas are introduced into infected herds or regions, where they are exposed to infected ticks and circulating virulent genotypes without prior immunity [2,10,49]. The prevalence of theileriosis is influenced by breed, as bovine breeds differ in tick resistance and innate susceptibility to infection; native cattle tend to have significantly lower prevalence than exotic breeds and crossbreeds [25,114]. Furthermore, multivariable analysis demonstrated that the risk of *T. orientalis* infection is higher in mixed-breed cattle and in animals over three years of age [159].

The published evidence supporting the occurrence of *T. orientalis* in each country is summarised in Supplementary Table S1. The table distinguishes molecular detection in vertebrate hosts from clinical outbreaks, historical serological or microscopic evidence, and molecular detection restricted to ticks or other haematophagous arthropods. The recognised host-vector range of bovine piroplasms has broadened following the detection of *T. orientalis* DNA within ixodid bat ticks [161]. These findings suggest that non-conventional hosts and their associated ectoparasites may contribute to parasite maintenance and dispersal in the environment, indicating a more complex epidemiological network than previously recognised [161].

Although *T. orientalis* and related genotypes have been detected in domestic bovids, wild ruminants and ticks associated with non-conventional hosts, the epidemiological significance of wildlife remains uncertain [8,12,136,161]. Most available evidence is based on cross-sectional molecular detection and does not demonstrate reservoir competence, sustained transmission to cattle or the direction of transmission between livestock and wildlife [49,136,161]. As the currently recognised significance of *T. orientalis* is primarily veterinary, its One Health relevance lies mainly in the shared livestock–wildlife–vector environment and in the need for coordinated surveillance across these compartments [8,12]. Paired longitudinal sampling of livestock, wildlife hosts and ticks, together with genotype comparison, is required to determine whether wildlife contributes to parasite maintenance or mainly reflects spillover from infected livestock [49,136,161].

Climate and environmental change may influence the epidemiology of oriental theileriosis indirectly by modifying the survival, development, seasonal activity and geographic suitability of competent tick vectors [49,59]. For *Haemaphysalis longicornis*, a modelling study conducted in New Zealand projected that areas climatically suitable for the tick could expand under future climate scenarios [162]. A separate model assessed current environmental suitability for *T. orientalis* transmission but did not evaluate future climate scenarios or observed disease incidence; its results should therefore not be interpreted as direct evidence that climate change is increasing the occurrence of oriental theileriosis [163]. Disease emergence also depends on the establishment and abundance of competent vector populations, the movement of infected carrier cattle, circulating parasite genotypes, host susceptibility, enzootic stability, land use and herd management [49,101]. Climate change should therefore be regarded as a modifying factor within a broader ecological and epidemiological system, rather than as an independent or sufficient explanation for the geographic expansion of oriental theileriosis.

Comparisons of prevalence among regions should be interpreted cautiously because the available studies differ substantially in sampling strategy, host selection, season, diagnostic method and molecular target. Cross-sectional PCR surveys provide evidence of parasite circulation but cannot determine the timing of infection, duration of carriage or causal relationship with clinical disease. Similarly, studies based on ticks may indicate local exposure but do not establish the prevalence of infection in cattle or confirm vector competence. These methodological differences probably account for part of the variation reported among countries and production systems.

## 7. Pathogenesis, Clinical Manifestations and Subclinical Infections

Current evidence supports a multistep model of pathogenesis in which parasite replication within erythrocytes induces structural and metabolic alterations, oxidative injury and enhanced erythrocyte clearance. These processes result in predominantly extravascular haemolytic anaemia and reduced oxygen-carrying capacity, while inflammatory dysregulation may amplify tissue injury and systemic clinical manifestations. However, the relative contribution of each mechanism has not yet been quantified experimentally.

### 7.1. Parasite–Erythrocyte Interactions

The initial pathological events are centred on the interaction between the parasite and the infected erythrocyte.

*Theileria species* are broadly classified into transforming and non-transforming groups based on their capacity to transform host leucocytes [3]. The evolution of this transforming capacity has been accompanied by marked changes in genome composition, including the acquisition or expansion of gene families considered critical to host–cell transformation [3,164]. Unlike transforming species, which induce lymphocyte proliferation in a neoplasia-like manner via the schizont stage [13,91,164], the main pathogenic effect of *T. orientalis* is the destruction of erythrocytes by intraerythrocytic piroplasms [2,13]. Schizonts can be detected transiently in lymph nodes, spleen and liver of infected cattle approximately 10 days after sporozoite inoculation [12]. However, schizont-infected cells are not commonly found in peripheral blood and are not typically associated with major pathogenic effects [12]. Although the pathogenic processes underlying *T. orientalis* infection remain incompletely understood [13], proposed mechanisms of erythrocyte destruction include direct lysis during merozoite egress, oxidative damage to erythrocyte membranes, and enhanced removal of parasitised or altered erythrocytes by the mononuclear phagocyte system [52,165,166]. The *T. orientalis* microneme–rhoptry protein (ToMRP), which is expressed during key stages of the erythrocytic cycle, was shown to bind band 3, a major component on the bovine erythrocyte membrane, suggesting a plausible molecular mechanism for parasite–erythrocyte interaction during invasion and/or egress [165]. Additionally, a thiol protease with haemoglobin-binding activity has been identified, which may contribute to parasite survival within erythrocytes [52]. The piroplasm surface protein p23 has also been characterised as an immunogenic heparin-binding protein; recombinant p23 binds heparin on glycoarrays, supporting a potential role in merozoite attachment and invasion, although this evidence is currently based on in vitro binding assays rather than direct erythrocyte-binding confirmation [96,167].

These molecular interactions provide plausible mechanisms for erythrocyte invasion and alteration, although their direct contribution to red-cell destruction in vivo remains incompletely demonstrated.

### 7.2. Mechanisms of Anaemia and Erythrocyte Clearance

Anaemia appears to result from the combined effects of direct parasite-associated injury, oxidative alteration of erythrocytes and enhanced extravascular clearance. The resulting anaemia is driven by high parasitaemia associated with the first wave of asexual reproduction, peaking approximately 2–3 months post-infection [91].

The pathogenesis of oriental theileriosis is characterised by extravascular haemolysis [46,166]. In this context, anaemia is thought to arise mainly from the splenic clearance of parasitised, damaged or immunologically altered erythrocytes, rather than from extensive intravascular lysis directly caused by parasite egress [168]. Splenic macrophage activation leads to the removal of both infected and uninfected erythrocytes from circulation, with denaturation of blood plasma postulated to further reduce erythrocyte survival [169,170]. Additionally, oxidation of haemoglobin to methaemoglobin has been documented experimentally in anaemic cattle infected with *T. orientalis*, with methaemoglobin concentration increasing in parallel with the onset and severity of anaemia. These oxidative alterations may reduce erythrocyte stability and promote recognition and removal by splenic macrophages. Together with oxidative bursts from phagocytic cells, this supports the involvement of oxidative erythrocyte damage in the pathogenesis of anaemia, potentially promoting the clearance of altered erythrocytes by the reticuloendothelial system [171,172]. Notably, erythrocyte destruction in *T. orientalis* can occur in the absence of immunoglobulins or complement activation, distinguishing it from classical antibody- or complement-mediated lysis observed in other haemoparasite infections [9,170,173].

Therefore, although direct erythrocyte lysis may contribute to red-cell loss, the available evidence suggests that oxidative injury and splenic erythrophagocytosis are central mechanisms underlying the progressive, predominantly extravascular anaemia associated with oriental theileriosis.

### 7.3. Inflammatory and Systemic Consequences

The consequences of erythrocyte loss extend beyond anaemia, as reduced oxygen delivery and inflammatory dysregulation may contribute to systemic and multiorgan disease. In affected cattle, intraerythrocytic piroplasms are associated with haemolytic anaemia and systemic disturbance, including haematobiochemical abnormalities, such as hyperbilirubinaemia, increased liver enzyme activities and azotaemia, alongside oxidative stress characterised by enhanced lipid peroxidation and reduced antioxidant capacity [25]. Cytokine changes may amplify this pathological process, although the available evidence remains observational and suggests immune dysregulation rather than a uniform systemic pro-inflammatory response. In naturally infected cattle, lower circulating interferon-gamma (IFN-γ) concentrations have been reported in *T. orientalis*-positive animals under grazing conditions [174]. More recently, clinically affected cattle showed significantly increased expression of interleukin-6 and interleukin-1β (IL-6 and IL-1β), with parasite burden positively correlated with both cytokines, whereas tumour necrosis factor-alpha (TNF-α) did not increase significantly and showed a non-significant downward trend [175]. These findings suggest that inflammatory activation may accompany macrophage-mediated erythrocyte clearance and contribute to systemic or hepatic dysfunction, although these relationships have not been demonstrated experimentally. In severe or fatal cases, multi-organ pathology is common, presenting as massive pulmonary oedema with froth in the trachea, abomasal ulceration, epicardial and endocardial haemorrhage and haemorrhagic duodenitis, occasionally accompanied by abortion or foetal loss [176]. Increased IL-6 and IL-1β expression may reflect macrophage activation and inflammatory amplification, whereas reduced IFN-γ suggests that the immune response is dysregulated rather than uniformly enhanced. Nevertheless, a causal link between these cytokine alterations, erythrocyte clearance and tissue injury has not yet been established.

### 7.4. Clinical Expression, Immunity and Determinants of Severity

Multiple outbreaks of oriental theileriosis associated with high morbidity and mortality have been reported, chiefly in the Asia-Pacific region (Australia and New Zealand), and more recently in the USA and India; reports from Africa and Europe indicate broader geographic occurrence with variable clinical impact [3,10,14,15,16,20,65,73,99,176,177,178,179,180]. Outbreak severity is strongly influenced by the predominant genotype and disease is typically associated with the Ikeda and Chitose genotypes, specifically Chitose A [181], as well as types 4 and 7 [14,15,99,125,176,179,180,182]. Approximately 1–6% of animals infected with the Ikeda genotype develop severe disease [15,183], and the degree of anaemia is directly dependent on the level of parasitaemia [184]. Recent experimental infections with the USA isolate of *T. orientalis* Ikeda genotype have demonstrated significant reductions in haematocrit and erythrocyte counts during acute infection, providing quantitative evidence of the haematological impact of this genotype [185]. Fatal cases have also been described in buffaloes in India associated with the N2 genotype [186]. In transforming *Theileria* species, protective immunity is mainly mediated by cytotoxic T cells, and antigenic diversity may contribute to immune evasion by reducing T-cell recognition of infected cells. In contrast, the immune mechanisms involved in non-transforming species such as *T. orientalis* remain poorly characterised [3]. Clinical signs intensify with increasing parasitaemia, correlating with haematological alterations [46]. Even for the Buffeli genotype, higher parasitaemia has been associated with clinical disease. Clinically affected cows had 3–22 piroplasms per 1000 erythrocytes and parasite loads ranging from $6.9\;\times \;10^{2}$ to $4.5\;\times \;10^{3}$ parasites/μL of blood, whereas apparently healthy infected cows had 0–1 piroplasms per 1000 erythrocytes and parasite loads ranging from $2.6\;\times \;10^{2}$ to $5.7\;\times \;10^{2}$ parasites/μL of blood [187]. Haematological alterations are evident in apparently healthy cattle, although they are relatively mild compared with clinically diseased animals [46]. Healthy animals may show a statistically significant reduction in erythrocyte parameters, especially in the warm season [157]. Haematological abnormalities are characterised by significant decreases in red blood cell count, haemoglobin concentration, and packed cell volume; leucocyte and platelet parameters may also be affected, depending on disease severity [19,46,159,166,185]. However, an increase in mean corpuscular volume (MCV) and eosinophil count are often observed and these fluctuations in erythrocyte and leucocyte parameters are directly associated with the degree of parasitaemia [46,158,159].

In calves aged < 6 months, haematological changes appeared less severe than in adult dairy cattle [166]. Passive immunity appears to confer only limited protection in young calves, as high infection prevalence and peak parasitaemia have been reported during the first weeks of life, particularly around 6–9 weeks of age, coinciding with reduced packed cell volume [49,86,188]. Seroconversion to the MPSP antigen occurs approximately 14 days after the onset of patency and humoral responses persist for at least 11 weeks post-infection [9,168]. Importantly, MPSP-specific antibody responses are significantly more frequent in clinically anaemic animals (approximately 89%) compared with subclinical carriers (approximately 45%), and seroconversion is strongly correlated with both parasite load and the Ikeda genotype [168]. This differential seroconversion rate underscores the limited reliability of serological assays for surveillance in subclinical infections and reinforces the superiority of molecular methods for carrier detection [168].

Infection with *T. orientalis* appears to have minimal impact on platelet dynamics, potentially masked by compensatory mechanisms; thus, thrombocytopenia is not characteristic of *T. orientalis* infection [46]. Reported clinical features include weakness, reluctance to walk, pyrexia, lethargy, lacrimation, nasal discharge, anaemia, pale mucous membranes, tachycardia, tachypnoea, swollen lymph nodes, icterus, anorexia, haemoglobinuria, abortion, hypogalactia, and death, particularly in periparturient animals and juveniles [25,46,91,99,101,176]. Atypical neurological signs have been associated with Chitose type B [99] with a higher incidence of cases in animals aged 3–5 years, predominantly during the periparturient period [25], reaching 5–10% mortality [15,16,54,99]. In post-outbreak investigations, oriental theileriosis was found to primarily affect beef cows older than two years during the pre-partum period, with outbreaks associated with abortion and cow mortality [102]. Combined infections with different *T. orientalis* genotypes may contribute to parasite persistence by simultaneously presenting multiple targets to the immune system [8]. The absence of cross-protection between genotypes is underscored by repeated infections with different types in susceptible cattle, although evidence regarding the association between disease severity and mixed-genotype infections remains limited [99]. Mixed infections have been reported among the following genotype combinations: Ikeda and type 7; Ikeda, Chitose and Buffeli; Chitose and Buffeli; and Ikeda and Buffeli [99,101,189]. In mixed infections, the Ikeda genotype is consistently detected first, with a shorter pre-patent period, as early as 11 days post-exposure to infected vectors and reaches its parasitaemic peak before the other genotypes [9]. A subsequent decline in Ikeda is followed by a progressive rise in Chitose, suggesting an immune-driven genotype switching mechanism, possibly reflecting the host’s selective immune pressure against the dominant genotype [181]. This temporal pattern has important clinical implications, as the initial Ikeda-driven parasitaemia appears responsible for the most severe haematological deterioration, including the earliest and steepest drops in packed cell volume [9,181].

Regarding co-infections with other pathogens, available evidence suggests that their effects on clinical expression are not uniform and may depend on the pathogen combination and epidemiological context. Cattle chronically infected with *T. orientalis* exhibited lower peak *Anaplasma marginale* parasitaemia, a longer time to reach peak parasitaemia and a reduced requirement for anaemia treatment, suggesting that co-infection may modulate the susceptibility to and clinical expression of anaplasmosis [190]. Because no serological cross-reactivity was detected between the two agents, antibody-mediated cross-protection was considered unlikely, and the authors suggested that this effect may involve non-antigen-specific cell-mediated immune mechanisms [190,191]. A possible interference phenomenon has also been described between *T. orientalis* and haemotropic *Mycoplasma* spp. (haemoplasmas), with single infections being more frequent than co-infections, and anaemia being significantly milder in co-infected animals [192]. However, this proposed interaction was not confirmed in a subsequent study, suggesting that such effects may vary according to management system, host population and epidemiological context [193,194]. In contrast, co-infections involving *Babesia ovata* and *T. orientalis* have been associated with the clinical development of anaemia [195,196,197]. Overall, these findings indicate that co-infections with other haemoparasites may modulate the clinical expression of *T. orientalis* infection, but their effects appear to be pathogen-specific and context-dependent rather than uniformly associated with increased virulence [107].

### 7.5. Subclinical Infections and Carrier State

Subclinical infection and persistent carriage represent an important component of the epidemiology and pathogenesis of *T. orientalis*, because infection may persist despite the resolution or absence of overt clinical disease.

Asymptomatic infections with *T. orientalis* have been reported in goats, sheep, buffaloes, Cape buffaloes, yaks and gayal [8,108,117,198,199,200,201,202,203]. The high proportion of subclinical carriers suggests widespread regional dissemination of *Theileria* infection, often undetected due to the absence of clinical signs [46]. Countries such as Italy report high prevalence with silent circulation, particularly in grazing animals [129]. Recovered animals remain asymptomatic carriers with low parasitaemia that can be detected by PCR for at least 30 months [91] and some authors have suggested lifelong persistence [91,103,105,106]. As piroplasms persist, relapses of disease may occur under stress conditions such as gestation, lactation, transport or by abrupt environmental or management changes [13,46]. Despite high antibody titres against MPSP in persistently infected cattle, recurrent parasitaemia is frequently observed, suggesting that humoral immunity against MPSP is insufficient to fully control infection [3].

Overall, the available evidence supports a multihit pathophysiological model. Parasite replication and parasite–erythrocyte interactions initiate red-cell alterations; oxidative damage and methaemoglobin formation impair erythrocyte function and survival; and splenic clearance of infected and apparently uninfected erythrocytes drives predominantly extravascular haemolytic anaemia. The resulting reduction in oxygen delivery, together with inflammatory dysregulation, may then contribute to organ dysfunction and clinical disease. However, the relative importance of direct parasite-induced lysis, oxidative membrane damage, erythrophagocytosis and cytokine-mediated injury remains to be established experimentally.

## 8. Productive and Economic Impact

Theileriosis remains a major constraint to cattle production, causing substantial mortality and production losses, particularly when associated with *T. annulata* and *T. parva* [12]. Within the non-transforming bovine *Theileria* group, *T. orientalis* is also increasingly recognised as economically relevant, with the most extensively quantified production losses reported in Australia and New Zealand; by contrast, evidence from Japan indicates that infection may have little detectable effect under intensive housing conditions [8,204]. However, the magnitude of losses experienced by any given region depends on multiple interacting factors, including herd density, predominant farming system, tick ecology, cattle immunity and cattle movement patterns [205].

Rather than being uniformly economically important across all endemic regions, oriental theileriosis appears to cause measurable economic losses mainly in clinically affected or recently exposed herds, particularly through reduced milk yield, impaired liveweight gain and mortality [105,106,183]. In this context, some authors have described the disease as one of the most economically important bovine diseases in humid tropical regions [2,10].

The principal economic losses associated with oriental theileriosis include abortions, significant reductions in milk yield and alterations in milk composition, and high morbidity and mortality [10]. These losses are most pronounced during clinical outbreaks, particularly in endemically unstable regions or following the introduction of naïve animals. In contrast, in chronically or subclinically infected herds in endemic settings, the effects on reproduction appear to be minimal: no detectable changes in reproductive parameters were reported in infected dairy cows [103]. Similarly, no differences were observed in the occurrence of concomitant diseases when comparing *T. orientalis*-infected and uninfected animals [204]. Although transient effects on libido may reduce conception rates, the overall impact of *T. orientalis* (Ikeda) infection on bull fertility appears to be negligible [206]. However, while some authors also report no productivity differences when comparing infected and uninfected cows [204], multiple studies have reported milk-production losses or decreased live weight gain associated with *T. orientalis* infection [103,105,106,183,204,207,208], although the reported impact remains inconsistent across studies.

In the Japanese study, 325 of 471 cows sampled during the dry period were PCR-positive (69.0%). Nevertheless, infected and uninfected cows had mean 305-day milk yields of 10,066 and 9650 kg, respectively (*p*-value = 0.11), and mean intervals from calving to conception of 110.9 and 104.2 days, respectively (*p*-value = 0.40), with neither difference reaching statistical significance [204].

Significant reductions in milk yield, milk fat percentage and milk protein percentage were reported at 100 and 305 days in lactation when comparing clinically affected *T. orientalis*-infected cows with negative, clinically healthy cows (uninfected controls) [106]. At 100 days of lactation, clinically affected PCR-positive cows produced 288 L less milk, 16.8 kg less milk fat and 12.6 kg less milk protein than clinically healthy PCR-negative cows. At 305 days, the corresponding deficits were 624 L of milk, 42.9 kg of milk fat and 26.0 kg of milk protein per cow [106]. Based on milk prices at the time of the study, the 624 L deficit represented an estimated loss of AUD 202 per severely affected cow. The total first-year loss associated with 16 severely affected cows and the death of one cow was estimated at AUD 5035, including AUD 1800 attributed to the death [106].

When positive cows with mild clinical signs were compared against negative cows in the same study, there were few differences in milk fat and protein at 100 days in lactation, and by 305 days, only milk fat was significantly affected [106]. Specifically, mildly affected cows produced 13.6 kg less milk fat and 8.6 kg less milk protein at 100 days, whereas at 305 days only the 21.2 kg reduction in milk fat remained statistically significant [106].

These findings contrast with another study in which prevalence was not associated with milk yield, although incident infections were associated with losses in energy-corrected milk [103]. On one New Zealand farm, cows that became infected during the study produced 266 kg less energy-corrected milk than uninfected cows (95% CI: 82–450 kg), corresponding to approximately 20 kg of milk solids. By contrast, cows that were already PCR-positive at the beginning of the study, representing predominantly chronic infections, showed no detectable reduction in milk production or reproductive performance [103].

Other authors reported effects extending beyond lactation, with infection being associated with reduced weight gain that was not recovered after six months [105]. In a longitudinal Australian study of 30 introduced beef weaners, the initial parasitaemic wave associated with the Ikeda and Chitose genotypes resulted in an estimated 20 kg deficit in weight gain, which had not been recovered six months after introduction [105].

The apparent disagreement among studies partly reflects differences in the populations and infection stages being compared. Studies involving clinically affected or recently infected cattle are more likely to identify production losses than studies comparing persistently PCR-positive, clinically healthy carriers with PCR-negative animals. Furthermore, several estimates originate from individual herds or relatively small numbers of clinically affected animals, which limits their generalisability. Differences in parasite genotype and burden, breed susceptibility, production system, follow-up period and the definition of PCR-negative comparison groups may also contribute to the inconsistent findings. Failure to quantify parasite burden or determine the circulating genotype in some studies further complicates comparisons between infection status and productive outcome. Direct monetary estimates are particularly scarce and remain largely restricted to individual outbreaks or specific production systems, limiting extrapolation to other regions. Overall, the available evidence supports substantial losses during clinical outbreaks and incident infection, whereas the productive and economic consequences of persistent subclinical infection remain uncertain and require larger, longitudinal and genotype-informed studies.

## 9. Diagnosis

Several diagnostic methods are available for the detection of *T. orientalis*, ranging from microscopy and serology to molecular assays. While conventional approaches remain useful in clinically affected animals, molecular techniques are generally preferred for sensitive detection, species identification and genotype discrimination, particularly in subclinical, chronic or mixed infections [10,53]. Table 2 summarises the main methods according to their sensitivity, specificity, cost, ease of use and practical limitations.

Although the main diagnostic approaches for *T. orientalis* are summarised in Table 2, the following section focuses on multiplex hydrolysis-probe quantitative PCR (qPCR) because it combines parasite detection, genotype discrimination and parasite load quantification, thereby providing information with direct clinical and epidemiological relevance. A qPCR assay was developed to detect and quantify *Theileria orientalis* and to differentiate clinically relevant genotypes [209]. This assay allowed infection to be classified into clinically relevant categories: low-level infections with ≤15,000 MPSP gene copies/μL of blood, moderate-level infections with >15,000 to ≤300,000 MPSP gene copies/μL of blood, and high-level infections with >300,000 MPSP gene copies/μL of blood [209]. In the validation population, low-level infections were associated predominantly with subclinical carrier animals, whereas 95% of cattle with parasite loads above 300,000 MPSP gene copies/μL of blood showed clinical signs [209]. Nevertheless, these thresholds should be regarded as diagnostic guides rather than universal clinical cut-offs, as their interpretation may depend on assay design, genotype, packed cell volume, clinical status and herd context [209].

From a practical perspective, microscopy remains useful as a rapid and inexpensive first-line method in clinically affected cattle, particularly when moderate or high parasitaemia is expected. However, a negative blood smear does not exclude infection in subclinical or chronically infected animals. Conventional PCR is more appropriate for confirming low-level infection and detecting carriers, whereas qPCR additionally allows parasite-load estimation and, depending on the assay, genotype discrimination. These advantages must be balanced against the higher cost of real-time PCR equipment, reagents and controls, the need for trained personnel and its limited availability in some diagnostic laboratories. Serological methods are more suitable for assessing previous exposure or herd-level seroprevalence than for confirming active infection, because antibody detection does not indicate current parasitaemia, parasite burden or infecting genotype.

Therefore, clinically suspected cases should ideally be assessed by blood-smear examination and haematology, with molecular testing used for confirmation, whereas PCR-based methods are preferable for asymptomatic animals, carrier detection and herd-level investigations.

## 10. Control Strategies

### 10.1. Herd-Level Surveillance and Biosecurity

Control of *T. orientalis* infection requires an integrated herd-level approach, as transmission depends on the interaction between infected or carrier cattle, competent tick vectors and local management conditions [49,91]. In the absence of broadly effective curative or preventive tools, control relies mainly on surveillance, early detection, vector control and management of movements involving naïve cattle [9,91].

Preventive management centres on reducing the exposure of susceptible animals to infected vectors [105]. The introduction of susceptible cattle into infected herds may increase the risk of clinical outbreaks; therefore, newly introduced animals should be temporarily separated and assessed by clinical examination and PCR-based testing before being mixed with the resident herd [214]. Deliberate exposure of calves to the parasite during the period of passive maternal immunity has been proposed as a form of premunition; however, this approach remains controversial and is not widely practised [91]. Frequent sampling may be used to monitor the spread of *T. orientalis* within newly affected herds but may be less informative once a high prevalence and persistent carriage are established [215]. Detection of *T. orientalis* DNA in the blood meals of haematophagous insects may provide a non-invasive method for obtaining supplementary evidence of local parasite circulation [92]. However, because this detection may reflect infected host blood rather than parasite development within the arthropod, it should not replace direct testing of cattle and does not demonstrate vector competence [49].

Hygienic and sanitary measures are also crucial for preventing possible mechanical transmission and should include washing and disinfection of castration knives, single-use needles or, where impractical (e.g., mass vaccination), use of sharp needles changed regularly to minimise blood transfer [84].

### 10.2. Integrated Vector and Environmental Management

Tick control remains the cornerstone of prevention. However, studying the vector competence of different ixodid species is critical for effective prevention planning. Distinguishing proven vector transmission capacity from mere presence of the parasite within the tick is essential [49]. Tick control is challenging because ticks may have a wide host range and overlapping periods of activity among developmental life stages, while most of their life cycle is spent off the host in the environment [59]. Integrated tick management combines chemical and non-chemical measures, including strategic acaricide use, pasture and environmental management, biological control, and selection of tick-resistant animals, with the aim of reducing tick burdens and pathogen transmission while limiting acaricide use and selection pressure for resistance [216]. Commercial Bm86-based anti-tick vaccination has been incorporated into control programmes targeting *Rhipicephalus microplus* in some Latin American settings [217,218]. However, its protection is species-dependent, and no commercial vaccine is currently available against *Haemaphysalis longicornis*, limiting its immediate relevance to the principal vector systems associated with oriental theileriosis [217]. Given the increasing challenge of acaricide resistance, integrated and context-specific tick control strategies are increasingly recommended over reliance on chemical control alone [216,219]. However, their effectiveness may still be limited by reinfestation, off-host tick stages, treatment costs and the difficulty of implementing multiple measures consistently under commercial conditions [216,219].

A *T. orientalis*-free grazing management system was reported after a seven-year period without cattle grazing and the exclusive introduction of uninfected heifers [220]. The system also includes monthly anaemia screening based on red blood cell parameters and tick-control measures aimed at reducing exposure to *T. orientalis-*infected haematophagous vectors [220]. Although successful under the specific conditions studied, maintaining pasture without grazing for seven years and introducing only uninfected animals may not be economically or operationally feasible in most commercial systems, and its applicability to other epidemiological settings remains uncertain.

### 10.3. Treatment and Supportive Care

Therapeutic options for oriental theileriosis remain limited. Buparvaquone has been associated with remission of clinical signs and reductions in parasitaemia, particularly when administered during the early stages of disease; however, the available evidence for *T.* orientalis is based mainly on relatively small field studies and experimental infections [27,91,221]; however, it is not approved for use in some countries [222,223]. In naturally infected cattle (with genotype Ikeda), buparvaquone, either alone or combined with long-acting oxytetracycline, improved clinical status but did not eliminate infection, indicating that treated animals may remain carriers [221]. Similarly, experimental treatment of subclinically infected cattle produced only transient effects and failed to clear the Ikeda genotype [27]. Toltrazuril administered at the time of experimental infection did not prevent calves from becoming infected, while the evidence supporting tulathromycin, primaquine, halofuginone and other reported treatment combinations remains too limited and heterogeneous to support general recommendations [91,221,224], treatment with diminazene diaceturate had lower efficacy in animals with high parasitaemia [225]. Although, in vitro, chloroquine, quinine and pyrimethamine inhibited *T. orientalis* proliferation [226]*,* these findings have not been translated into demonstrated clinical efficacy in cattle.

Clinical management should therefore prioritise early detection and supportive care. Blood transfusion may be required in cattle with severe, life-threatening anaemia, although cost, availability and variable clinical outcomes may limit its use, while antimicrobial treatment should be reserved for animals with suspected secondary bacterial infections [9]. Grazing bovines treated preventively with ivermectin showed a lower frequency of *T. orientalis* infection and a smaller reduction in certain erythrocyte parameters during the grazing season, suggesting a benefit in preventing infection under field conditions [227]. Overall, currently available treatments may promote clinical recovery but cannot be relied upon to eliminate persistent infection.

### 10.4. Vaccine Prospects and Remaining Barriers

Vaccination has been explored both against *T. orientalis* and its principal vector, *Haemaphysalis longicornis*. However, no vaccines are currently available for either, despite ongoing efforts [91,217,228]. Attempts have been made to use recombinant proteins and peptides, particularly the major piroplasm surface protein (MPSP), as strategies for vaccine development [229]. These studies produced variable results: some formulations reduced parasitaemia or suggested partial protection, whereas others induced antibody responses without protecting cattle after challenge. Whole-blood preparations also raised biosafety concerns because of the potential transfer of other blood-borne pathogens [91].

The antigenic piroplasm-stage protein To ORF2 has also been proposed as a potential vaccine candidate. The protein was recognised by serum from an infected calf, and antibodies raised against recombinant To ORF2 recognised the native protein in piroplasm lysates and infected erythrocytes. However, its protective efficacy was not assessed, and immunogenicity alone does not demonstrate that an antigen can protect cattle against infection or clinical disease [230]. Given that the pathogenesis of this theileriosis is incompletely understood, vaccine development remains challenging. Nevertheless, the MPSP gene represents a promising target for a subunit vaccine, as genetic variability within MPSP is associated with differential virulence and includes determinants for both cellular and humoral immunity [3,13,168]. MPSP remains a biologically relevant candidate because it is abundant, immunodominant and expressed across the principal genotypes. Nevertheless, its genetic and antigenic variability may limit broad cross-protection, particularly in animals infected with multiple genotypes. The protective immune mechanisms against *T. orientalis* also remain incompletely defined, while previous MPSP-based vaccination studies have not generated consistent protection. Differences in vaccine formulation, challenge route and intensity, parasite genotype and the criteria used to define protection further complicate comparison among studies. These limitations help explain why promising antigen recognition has not yet resulted in a licenced vaccine [13,91].

Recent in silico approaches have also contributed to the identification of potential therapeutic targets for *Theileria* spp. [231]. A comparative proteomics and subtractive genomics study across *T. parva*, *T. annulata* and *T. orientalis* isolates identified conserved, parasite-specific and host non-homologous proteins with predicted druggability [231]. Among these, ubiquitin carboxyl-terminal hydrolase, phosphatidylinositol 3/4-kinase and DNA polymerase A were prioritised as promising anti-theilerial drug targets based on predicted essentiality, evolutionary conservation and structural suitability for ligand binding [231].

Anti-tick vaccination could provide a complementary approach by reducing tick feeding, reproduction and pathogen transmission. Candidate antigens identified in *H. longicornis*, including the ATAQ homologue HlATAQ and other genome-derived targets, remain at an early experimental stage [217,228].

Beyond conventional control measures, host genetic variability may hold future significance in the prevention of *T. orientalis* infection. Several studies suggest the existence of specific alleles associated with increased resistance or susceptibility; however, this approach requires further validation before it can be formally integrated into breeding programmes [232].

## 11. Conclusions

*T. orientalis* has shifted from being historically regarded as a benign or low-virulence parasite [9,10] to being increasingly recognised as a pathogen of clinical and economic importance, particularly in infections involving pathogenic genotypes such as Ikeda [2,8,14,15]. This review highlights that clinical outcomes are strongly influenced by parasite genotype, with the Ikeda and Chitose genotypes being primarily responsible for severe outbreaks [10,14,15,99,182]. Nevertheless, significant knowledge gaps persist: the pathogenic mechanisms driving erythrocyte destruction remain poorly understood [13]; vector competence has been experimentally confirmed for only a limited number of ixodid species [10]; and no licensed vaccines or fully effective treatments are available, with the carrier stage appearing to be lifelong and irreversible [91,221].

Future research should prioritise: (i) experimental validation of vector competence across tick species, particularly European species that have been implicated by field or molecular evidence [22,49,54]; (ii) development of effective vaccines, with MPSP-based antigens representing promising subunit vaccine candidates [3,9,168,229,233]; (iii) improved epidemiological surveillance in regions where prevalence, genotype distribution, vector associations and clinical relevance remain insufficiently characterised, including several European settings [22,49,234]; and (iv) assessment of the economic impact of subclinical infections on production parameters [103,106,204,235]. In the context of climate change and changing tick distributions, sustained investment in surveillance, genotyping, vector studies and integrated control strategies will be essential to reduce the global impact of oriental theileriosis [49,91,216].

## Figures and Tables

**Table 1:** Relationship between tick species (Ixodidae) and the current knowledge on their capacity to transmit *Theileria orientalis.*

Tick Species	Current Evidence of *T. orientalis* Vector Competence	Reference
*Amblyomma variegatum*	Presence of the agent	[67]
*Amblyomma coharens*	Presence of the agent	[67]
*Dermacentor nuttali*	Presence of the agent	[68]
*Dermacentor marginatus*	Presence of the agent	[69]
*Haemaphysalis bispinosa*	Possible transmission	[70]
*Haemaphysalis bancrofti*	Presence of the agent	[56]
*Haemaphysalis douglasi*	Possible transmission	[71]
*Haemaphysalis humerosa*	Experimental transmission	[35]
*Haemaphysalis longicornis*	Transmission	[54]
*Haemaphysalis mageshimaensis*	Presence of the agent	[72]
*Haemaphysalis megaspinosa*	Possible transmission	[71]
*Haemaphysalis punctata*	Possible transmission	[73]
*Haemaphysalis qinghaiensis*	Possible transmission of a *T. orientalis*-related *Theileria* spp.	[74]
*Hyalomma marginatum*	Presence of the agent	[69]
*Hyalomma detritum*	Presence of the agent	[75]
*Hyalomma excavatum*	Presence of the agent	[76]
*Hyalomma lusitanicum*	Presence of the agent	[69]
*Hyalomma scupense*	Presence of the agent	[77]
*Ixodes ricinus*	Presence of the agent	[78]
*Ixodes ovatus*	Possible transmission	[71]
*Ixodes persulcatus*	Possible transmission	[71]
*Rhipicephalus annulatus*	Presence of the agent	[79]
*Rhipicephalus bursa*	Presence of the agent	[69]
*Rhipicephalus decoloratus*	Presence of the agent	[80]
*Rhipicephalus evertsi*	Presence of the agent	[80]
*Rhipicephalus haemaphysaloides*	Presence of the agent	[70]
*Rhipicephalus microplus*	Transmission	[81]
*Rhipicephalus praetextatus*	Presence of the agent	[67]
*Rhipicephalus sanguineus*	Presence of the agent	[82]
*Rhipicephalus turanicus*	Presence of the agent	[83]

Note: “Presence of the agent” refers to the molecular detection (e.g., PCR) of *T. orientalis* DNA in ticks collected from cattle or the environment, without evidence that the tick can transmit the parasite to a vertebrate host. “Possible transmission” indicates that the tick species has been suggested as a potential vector based on field association, epidemiological evidence, or detection of the parasite in naturally infected ticks, but without controlled experimental confirmation of transmission. “Experimental transmission” indicates that the tick species has been shown, under experimental conditions, to transmit *T. orientalis* to a vertebrate host. “Transmission” refers to tick species reported in the literature as vectors of *T. orientalis*; although the strength of evidence varies among studies.

**Table 2:** Comparison of diagnostic methods for *Theileria orientalis.*

Method	Sensitivity	Specificity	Cost	Ease of Use	Notes	Reference
Microscopy (*Giemsa-*stained blood smear)	Low	Moderate	Very low	High	Ineffective for chronic carriers; unable to distinguish genotypes	[10]
Conventional PCR	High	High	Moderate	Moderate	Detects carriers; enables genotyping	[209]
qPCR (quantitative)	Very High	Very High	High	Moderate	Highly sensitive, quantifies parasite load	[209]
ddPCR	Very High	Very High	High	Low	High analytical precision; expensive and infrequently used for routine diagnostics	[210]
LAMP	High	High	Low/Moderate	High	Rapid; does not require a thermocycler; potentially suitable for rapid field diagnosis.	[211,212]
ELISA/IFAT	Moderate	Low/Moderate	Moderate	Moderate	Useful for prevalence studies; risk of cross-reactivity with other species.	[1,10,44]
Reverse Line Blot Hybridization (RLB)	Very High	Very High	Low	Moderate	Multi-species panel that detects co-infections, can be expanded and is suitable for surveillance.	[37,213]

Note: Sensitivity, specificity, cost and ease of use are presented as qualitative relative categories based on a narrative synthesis by the authors of the method-development studies, validation studies and reviews cited for each diagnostic approach. These categories do not correspond to predefined quantitative thresholds or to a single direct comparison of all methods. Diagnostic performance may vary according to sample quality, parasite burden, stage of infection, laboratory infrastructure, DNA-extraction procedure, primer or probe design and diagnostic target. Costs and ease of use are considered relative to routine veterinary diagnostic settings and may vary among laboratories and countries. Molecular assays also differ in their capacity for species identification, genotype discrimination and parasite-load quantification.

## Data Availability

No new data were created or analysed in this study. Data sharing is not applicable to this article.

## Supplementary Materials

The following supporting information can be downloaded at: https://www.mdpi.com/article/10.3390/ani16152348/s1, Table S1, Country-level evidence of *Theileria orientalis* occurrence, genotype distribution, clinical relevance and vector associations.

## Abbreviations

The following abbreviations are used in this manuscript:

ddPCR	Droplet digital polymerase chain reaction
DNA	Deoxyribonucleic acid
ELISA	Enzyme-linked immunosorbent assay
IFN-γ	Interferon-gamma
IFAT	Indirect fluorescent antibody test
IL-1β	Interleukin-1 beta
IL-6	Interleukin-6
ITS	Internal transcribed spacers
kDa	Kilodalton, a unit of molecular mass
LAMP	Loop-mediated isothermal amplification
MCV	Mean corpuscular volume
MPSP	Major piroplasm surface protein
PCR	Polymerase chain reaction
qPCR	Quantitative polymerase chain reaction
RLB	Reverse line blot hybridisation
rRNA	Ribosomal ribonucleic acid
TNF-α	Tumour necrosis factor-alpha
ToMRP	*T. orientalis* microneme–rhoptry protein
USA	United States of America
