# Supplementary material for: From Benign Parasite to Emerging Pathogen: A Review of Theileria orientalis in Ruminants
Source: Animals (Basel). 2026 Aug 1;16(15):2348. doi: 10.3390/ani16152348 (PMC13463525; doi:10.3390/ani16152348)
Supplement: Supplementary file 1 [file animals-16-02348-s001.zip › animals-4420644-supplementary.pdf]

Supplementary Materials:

Supplementary Table S1. Country-level evidence of *Theileria orientalis* occurrence, genotype distribution, clinical relevance, and vector association.

Country	Study area	Host or sample type	Evidence category	Diagnostic method	Sample size and prevalence	Genotype(s)	Clinical status or impact	Vector evidence	Reference
Canada	Eastern Ontario	Dairy cattle; 3-year-old Holstein cow	Molecularly confirmed clinical case in a vertebrate host	Blood-smear examination; 18S rRNA amplification and gene sequencing	Single clinical case; prevalence not assessed	Not determined; reported as *T. buffeli* complex	Pyrexia, diarrhoea, lameness, weight loss, marked anaemia, hypoproteinaemia and hyperbilirubinaemia	Vector not identified	[149]
		Dairy cattle; 3-year-old Holstein cow imported from the USA	Imported molecularly confirmed clinical case; no evidence of local transmission	Blood-smear examination; 18S rRNA PCR and sequencing; genotype confirmation by the Canadian Food Inspection Agency	Single clinical case; prevalence not assessed	Ikeda	Marked regenerative anaemia, pale mucous membrane, reduced appetite and milk production	Vector not identified	[148]

Country	Study area	Host or sample type	Evidence category	Diagnostic method	Sample size and prevalence	Genotype(s)	Clinical status or impact	Vector evidence	Referen ce
United States of America	Central Missouri	Beef cattle; naturally infected herd and two experimentally infected splenectomised calves	Molecularly confirmed clinical case and herd investigation; experimental blood transmission	Blood-smear examination, IFAT, DNA sequencing, transmission electron microscopy and experimental transmission	75 cattle examined; 22/75 (29%) parasitaemic and 26/75 (35%) seropositive	Not determined; reported as *T. buffeli*	Severe anaemia, lymphoid hyperplasia, dysproteinaemia and liver dysfunction in the index cow; mild anaemia in experimentally infected calves	Tick vector not determined	[151]
	Virginia	Beef cattle from several herds	Molecularly confirmed clinical outbreak in vertebrate hosts	Blood-smear examination; MPSP- and SSU rDNA-targeted PCR; bidirectional Sanger sequencing	Targeted outbreak investigation; sequences obtained from 10 cattle representing six herds; prevalence not estimable	Ikeda	Weakness, malaise, icterus and severe anaemia; seven cattle died in the index herd; persistent PCR positivity was observed in some cattle	*H. longicornis* occurred in the affected region; its competence for transmission of the US Ikeda isolate was subsequently demonstrated experimentally	[54,150]

Country	Study area	Host or sample type	Evidence category	Diagnostic method	Sample size and prevalence	Genotype(s)	Clinical status or impact	Vector evidence	Reference
Brazil	State of Pará, Amazon region	Apparently healthy Asian water buffaloes; six farms	Molecular detection in vertebrate hosts, with phylogenetic placement within the *T. orientalis* group	Nested PCR targeting the 18S rRNA gene and complete ITS region; sequencing and phylogenetic analysis	308 buffaloes; 13/308 positive (4.2%); positive animals occurred on 3/6 farms	Not formally assigned; sequences were closely related to *T. buffeli*, *T. orientalis* and *T. sinensis*	Animals were apparently healthy; 11/13 positive buffaloes originated from a farm with a history of lymphoproliferative disease, although causality was not established	Not assessed	[147]
	State of Pará, Amazon region; Marajó Island and mainland	Water buffaloes	Molecular detection and MPSP genotyping in vertebrate hosts	PCR amplification of the MPSP gene; sequencing and phylogenetic analysis	271 buffaloes; 39/271 positive (14.4%)	N1, N2 and N4; N4 was predominant and reported for the first time in Brazil	Clinical impact not assessed; the study focused on molecular occurrence and genetic diversity	Not assessed; *Rhipicephalus microplus* was identified as a possible candidate for future vector studies, not as a confirmed vector	[100]
Paraguay	Nine departments in the Eastern and Western regions	Cattle	Molecular detection and genotyping in vertebrate hosts	Pathogen-specific PCR; MPSP sequencing and phylogenetic analysis	326 cattle; 2.4% positive for *T. orientalis*	Chitose, Ikeda and Buffeli	Clinical status and productive impact not assessed; cross-sectional epidemiological survey	Not assessed	[146]

Country	Study area	Host or sample type	Evidence category	Diagnostic method	Sample size and prevalence	Genotype(s)	Clinical status or impact	Vector evidence	Reference
Bosnia and Herzegovina	Pale municipality, Sarajevo–Romanija region	Cattle from smallholder farms; whole-blood samples	Molecular detection in vertebrate hosts	Commercial piroplasm PCR, bidirectional sequencing and phylogenetic analysis	30 cattle from 10 farms; piroplasm DNA detected in 13/30 cattle (43%); positive cattle occurred on 6/10 farms (60%); *T. orientalis* confirmed by sequencing	Not determined	Clinical impact was not specifically evaluated; the findings indicated a possible enzootic focus	Not assessed	[134]
Croatia	Croatia; indigenous cattle cases	Clinically affected cattle; whole-blood samples	Molecularly confirmed indigenous clinical infection	PCR and sequence-based molecular identification	Case series involving three affected cows; population prevalence was not assessed	*T. orientalis*; genotype not determined	Indigenous clinical disease with fatal outcomes was reported, but the contribution of *T. orientalis* could not be separated fully from concurrent *Anaplasma* infections	Not assessed	[126]
France	Several locations in France	Cattle; whole-blood samples	Molecular detection in vertebrate hosts	PCR and sequence-based molecular characterisation	*T. buffeli* detected in 5 cows; overall bovine piroplasm prevalence: 8.8%	Not determined; reported as *T. buffeli*	Clinical status and productive impact were not reported	Not assessed	[127]

Country	Study area	Host or sample type	Evidence category	Diagnostic method	Sample size and prevalence	Genotype(s)	Clinical status or impact	Vector evidence	Reference
Greece	Macedonia region; 33 locations	Cattle sera and blood smears	Serological and microscopic evidence in vertebrate hosts	Indirect fluorescent antibody test and Giemsa-stained blood-smear examination	602 serum samples; 41.4% were seropositive to *T. orientalis*; blood smears were examined from 50 cattle	Not determined	Clinical impact was not assessed; interpretation of seropositivity is limited by possible cross-reactions	Not assessed in this study	[128]
Hungary	Mátranovák, Nógrád County, northeastern Hungary	Beef cattle; blood samples from a herd with clinical piroplasmosis	Molecular detection in vertebrate hosts, including a clinical case	Giemsa-stained blood smears; conventional PCR targeting the 18S rRNA gene; sequencing	Herd of 88 cattle; 85 cattle screened; *T. buffeli* detected in 4 cattle, approximately 4.7% of those screened	Not determined; reported as *T. buffeli*	One *T. buffeli*-positive cow had anaemia with a haematocrit of 22% and recovered following treatment; the other infected cattle were apparently healthy	*H. punctata* was found on the affected pasture and is a recognised potential vector, but transmission was not demonstrated in the study	[73]
Italy	Central and Northern Italy	Cattle blood and ticks collected from cattle	Molecular detection in vertebrate hosts and parallel tick investigation	Blood-smear microscopy, PCR, sequencing/BLAST analysis and serology	468 cattle; 21.6% were PCR-positive for piroplasms overall; 67 amplicons, corresponding to 17.0%, were assigned to the *T. sergenti/buffeli/orientalis* group	Not determined; *T. sergenti/buffeli/orientalis* group	Epidemiological survey; specific clinical or productive effects of infection were not assessed	127 ticks were examined; the *T. sergenti/buffeli/orientalis* group was not demonstrated in ticks, and vector competence was not established	[129]

Country	Study area	Host or sample type	Evidence category	Diagnostic method	Sample size and prevalence	Genotype(s)	Clinical status or impact	Vector evidence	Reference
Portugal	Mainland Portugal; northern, central, Lisbon and Tagus Valley, Alentejo and Algarve regions	Asymptomatic cattle; whole-blood samples	Molecular detection in vertebrate hosts	PCR followed by reverse line blot hybridisation (PCR–RLB)	1,407 cattle; *T. buffeli* detected in 10.1%; overall piroplasm prevalence: 36.8%	Not determined; reported as *T. buffeli*	Infections were detected in asymptomatic cattle; clinical and productive impacts were not assessed	Not assessed	[23]
Romania	Twenty counties in Romania	Ticks collected from cattle and other animal hosts	Molecular detection in ticks only	PCR and sequencing	852 ticks collected from 10 animal species; *T. buffeli* detected in 2 *Rhipicephalus bursa* samples collected from cattle	Not determined; reported as *T. buffeli*	Not applicable; direct infection or clinical disease in cattle was not demonstrated by this study	Parasite DNA was detected in *R. bursa*, but detection does not demonstrate vector competence	[130]
Russia	Moscow region	Holstein cattle; whole-blood samples	Molecular characterisation in vertebrate hosts	Full-length 18S rRNA PCR, sequencing and phylogenetic analysis	15 previously positive cattle were characterised; prevalence could not be estimated from the selected sample	*T. orientalis*/*T. buffeli*/*T. sergenti* group; Chitose-related lineage based on 18S rRNA phylogeny, but not confirmed by MPSP genotyping	Clinical status and productive impact were not reported	Not assessed	[131]

Country	Study area	Host or sample type	Evidence category	Diagnostic method	Sample size and prevalence	Genotype(s)	Clinical status or impact	Vector evidence	Referen ce
Serbia	Six locations, with positive cattle originating from Banatski Karlovac	Dairy cattle; blood samples and ticks collected from cattle and pastures	Molecular detection of a *T. orientalis*-related organism in vertebrate hosts	Conventional PCR followed by sequencing	185 cattle sampled; 135 blood samples tested molecularly; 5/135 (3.7%) were positive for piroplasm DNA	Benign *Theileria* spp.; sequences showed 100% identity to entries reported as *T. sergenti*, *Theileria* spp. and *T. buffeli* isolate HS252	Positive cattle showed no clinical signs attributable to theileriosis	Tick species were recorded, but ticks were not tested and vector involvement was not assessed	[132]
Spain	Mainly Northern Spain; samples from 79 farms	Cattle; whole-blood samples	Molecular detection in vertebrate hosts	PCR–RLB, blood-smear microscopy and haematology	263 cattle; *T. buffeli* detected in 42.6% of samples; overall RLB positivity: 54.0%	Not determined; reported as *T. buffeli*	Many RLB-positive and microscopy-negative cattle had haematological values within reference ranges, suggesting subclinical or chronic carrier infections	Not assessed	[24]

Country	Study area	Host or sample type	Evidence category	Diagnostic method	Sample size and prevalence	Genotype(s)	Clinical status or impact	Vector evidence	Reference
Switzerland	Canton of Grisons (Graubünden), alpine Switzerland	Dairy cattle; whole-blood samples from a herd affected by haemolytic anaemia	Molecular detection in vertebrate hosts during a multi-pathogen clinical outbreak	PCR and partial 18S rRNA gene sequencing	286 cattle examined; *Theileria*-specific prevalence was not reported separately	*T. buffeli/sergenti/orientalis* group; MPSP genotype not determined	Fatal haemolytic anaemia occurred in a herd with multiple concurrent infections; *A. marginale* was considered the main cause, while coinfections may have aggravated disease	Vector not identified; local transmission and epidemiology remained undetermined	[133]
Turkey	Cattle herds in Turkey	Apparently healthy cattle; whole-blood samples	Molecular detection and MPSP genotyping in vertebrate hosts	Genotype-specific PCR and MPSP sequencing	63 cattle; Buffeli alone detected in 46.0%, Chitose alone in 11.1%, and mixed Buffeli–Chitose infections in 17.5%	Chitose and Buffeli	Cattle were clinically healthy; clinical and productive effects were not assessed	Vector competence was not investigated	[125]
United Kingdom	United Kingdom livestock collection site	Blood-fed *Culiseta annulata* mosquitoes; blood meals originated predominantly from cattle	Indirect molecular evidence from arthropod blood meals	Pan-piroplasm PCR and sequencing	16 blood-meal samples (15.2%) were positive; most identified blood meals were from cattle	*T. orientalis* group; genotype not determined	Clinical disease in cattle was not assessed; the study provided indirect evidence of parasite circulation in livestock	Detection in mosquito blood meals does not demonstrate mosquito transmission or vector competence	[92]

Country	Study area	Host or sample type	Evidence category	Diagnostic method	Sample size and prevalence	Genotype(s)	Clinical status or impact	Vector evidence	Reference
Algeria	Bouira and Tizi-Ouzou, northern Algeria	Cattle, sheep and goats; whole-blood samples	Molecular detection and phylogenetic characterisation in vertebrate hosts	PCR targeting the mitochondrial Cox3 and Tams1 genes, followed by sequencing	149 ruminants; 19/149 (12.75%) were positive for *Theileria* spp., all in cattle. Seventeen were identified as *T. annulata* and two as *T. orientalis*	Novel *T. orientalis* Cox3 haplotype; MPSP genotype not determined	Clinical status and productive impact were not assessed	Not assessed	[137]
Egypt	Four governorates in northern Egypt, including Kafr El-Sheikh and Gharbia	Asymptomatic cattle; whole-blood samples	Molecular detection and MPSP genotyping in vertebrate hosts	PCR targeting the MPSP gene, sequencing and phylogenetic analysis	500 asymptomatic cattle; 44/500 positive (8.8%)	Type 1, Chitose and type 2, Ikeda	All sampled cattle were asymptomatic; clinical and productive effects were not evaluated	Tick infestation and absence of acaricide use were identified as risk factors, but vector competence was not investigated	[138]
Morocco	Rabat–Salé–Kénitra region; nine locations and 117 farms	Cattle blood and ticks collected from cattle	Molecular detection in vertebrate hosts, with a parallel tick survey	Conventional PCR targeting the 18S rRNA gene, sequencing and phylogenetic analysis	508 cattle; 149/508 (29.3%) were positive for *Theileria*/*Babesia* spp. Among 131 successfully sequenced samples, 24 contained *T. buffeli* and 18 contained *T. orientalis*	Species identified by 18S rRNA; MPSP genotypes not determined	Epidemiological survey involving both clinically healthy and sick cattle; the specific clinical impact *of T. orientalis* was not established	3,394 ticks from eight Hyalomma and Rhipicephalus species were collected, but ticks were not tested for the pathogens and vector competence was not assessed	[139]

Country	Study area	Host or sample type	Evidence category	Diagnostic method	Sample size and prevalence	Genotype(s)	Clinical status or impact	Vector evidence	Reference
Tunisia	Different cattle-rearing areas in Tunisia	Cattle blood and ticks collected from cattle	Molecular detection in vertebrate hosts, with a tick-distribution survey	Blood-smear microscopy, PCR–RLB and molecular identification	278 cattle; 104/278 (37.4%) were positive for piroplasms by microscopy; *T. buffeli* was detected in 39.2% of samples by PCR–RLB	*T. buffeli/sergenti/orientalis* group; MPSP genotype not determined	Clinical impact attributable specifically to *T. orientalis* was not assessed	Tick species and distributions were recorded, but competence for transmission of *T. orientalis* was not demonstrated	[135,143]
Cameroon	Western Highlands of Cameroon	Zebu cattle; whole-blood samples	Molecular detection in vertebrate hosts	Nested PCR targeting the 18S rRNA gene, followed by reverse line blot hybridisation	162 cattle; *T. orientalis* detected in 5.55%; overall piroplasm prevalence was 82.1%	Not determined	The authors interpreted positive cattle as probable subclinical carriers; direct clinical effects were not assessed	No tick vector was tested experimentally; the responsible vector remains undetermined	[140]
Ethiopia	Different regions of Ethiopia	Apparently healthy local-breed cattle; whole-blood samples	Molecular detection and MPSP genotyping in vertebrate hosts	Conventional PCR targeting the MPSP gene, followed by sequencing	232 cattle; 5/232 positive (2.2%)	Buffeli, in 3/232 and type 5, in 2/232	Apparently healthy cattle; clinical and productive impacts were not assessed	Not assessed	[64]

Country	Study area	Host or sample type	Evidence category	Diagnostic method	Sample size and prevalence	Genotype(s)	Clinical status or impact	Vector evidence	Reference
	Southwestern Ethiopia	Cattle; whole-blood samples	Molecular detection in vertebrate hosts	PCR followed by reverse line blot hybridisation	392 cattle; 203/392 positive for *T. orientalis* (51.8%)	Not determined in the study	High frequency of infection and coinfection; clinical impact attributable specifically to *T. orientalis* was not assessed	Not assessed	[141]
Kenya	Ngong and Machakos dairy farms	Apparently healthy dairy cattle; whole-blood samples	Molecular detection and genetic characterisation in vertebrate hosts	Nested PCR targeting the 18S rRNA and MPSP genes, followed by sequencing	192 cattle; *T. orientalis* detected in 3 cattle, corresponding to 3/192 overall (1.6%) and 3/154 in Ngong (1.9%)	Buffeli and type 5	All sampled cattle appeared clinically healthy; positive animals had mixed infections with other haemoparasites	Vector competence was not assessed	[144]
Nigeria	Makurdi and Abinsi, Benue State	Cattle; whole-blood samples	Molecular detection in vertebrate hosts	Semi-nested PCR targeting the 18S rRNA gene, species-specific PCR and sequencing of representative amplicons	160 cattle; 18/160 (11.25%) positive for *T. orientalis*, including single and mixed infections	Not determined	Clinical status was not systematically evaluated; the study focused on prevalence and risk factors	Vector competence was not assessed; lack of acaricide use and free-range management were associated with infection risk	[142]

Country	Study area	Host or sample type	Evidence category	Diagnostic method	Sample size and prevalence	Genotype(s)	Clinical status or impact	Vector evidence	Reference
Burundi	Burundi; precise study location not reported in the available abstract	Cattle involved in an outbreak of bovine theileriosis	Historical clinical outbreak with morphological and serological identification in vertebrate hosts	Morphological examination and serological characterisation	Outbreak investigation; sample size and prevalence not reported in the available abstract	Not determined	*T. orientalis* was associated with clinical signs during an outbreak of bovine theileriosis	*Amblyomma variegatum* was suspected as the local vector; vector competence was not experimentally demonstrated	[65]
South Africa	Hluhluwe–iMfolozi Game Park and Addo Elephant Game Park	African buffalo (*Syncerus caffer*); whole-blood samples	Molecular detection and phylogenetic characterisation in a wildlife host	PCR amplification, cloning and sequencing of full-length 18S rRNA and complete ITS regions	Selected previously positive buffalo samples; prevalence could not be estimated from this study	Novel *T. buffeli*-like and *T. sinensis*-like genotypes	Clinical impact was not assessed; the study raised the possibility that African buffaloes could act as reservoir hosts	Vector not assessed	[136]
	Raymond Mhlaba Local Municipality, Eastern Cape Province	Ticks collected from free-ranging domestic animals	Molecular detection in ticks only	Genus-specific PCR, sequencing and phylogenetic analysis	962 ticks; *Theileria* DNA detected in 10/962 ticks (approximately 1%); nine sequences clustered within the *T. orientalis* group	*T. orientalis* group; MPSP genotype not determined	Not applicable; infection or clinical disease was not demonstrated directly in vertebrate hosts	DNA was detected mainly in *Rhipicephalus* spp.; this does not demonstrate vector competence	[66]

Country	Study area	Host or sample type	Evidence category	Diagnostic method	Sample size and prevalence	Genotype(s)	Clinical status or impact	Vector evidence	Reference
Kazakhstan	Turkistan and Zhambyl Provinces, southern Kazakhstan; 40 villages in nine administrative districts	Adult cattle; whole-blood samples	Molecular detection and MPSP genotyping in vertebrate hosts	Species-specific PCR targeting the MPSP gene, followed by sequencing	766 cattle; 33.3% positive for *T. orientalis*; positive cattle were detected in 31/40 villages	Chitose and Buffeli; 2 of 14 characterised sequences belonged to Chitose and 12 to Buffeli	Cross-sectional epidemiological survey; clinical signs and productive impact were not evaluated	Vector species and vector competence were not investigated	[118]
Kyrgyzstan	Eight locations around the Chu Valley and Issyk-Kul Lake	Apparently healthy cattle; whole-blood samples	Molecular detection in vertebrate hosts, followed by MPSP genotyping	PCR–reverse line blot for species detection; MPSP PCR, SSCP, sequencing and genotype-specific PCR	454 cattle; 149/454 positive for *T. orientalis* (32.8%). The 149 positive samples were subsequently genotyped	Buffeli, alone in 26.2%; Chitose A, alone in 2.0%; mixed Buffeli–Chitose infection in 71.8%; Ikeda not detected	Clinical infection was not evaluated; the sampled cattle were apparently healthy	Vector competence was not investigated	[116,119]
Bangladesh	Jhenaidah, Bogura, Sirajganj, Bandarban and Naikhongchari	Cattle, gayals and goats; whole-blood samples	Molecular detection and MPSP genotyping in vertebrate hosts	Species-specific PCR, MPSP sequencing and phylogenetic analysis	276 animals; overall *T. orientalis* prevalence 46.01%. Prevalence in cattle was 67.24%	Types 5 and 7	Epidemiological survey; specific clinical effects were not evaluated. Coinfections were frequent, particularly with *Babesia bigemina*	The study discussed exposure to *Haemaphysalis bispinosa*, but vector competence was not tested directly	[117]

Country	Study area	Host or sample type	Evidence category	Diagnostic method	Sample size and prevalence	Genotype(s)	Clinical status or impact	Vector evidence	Referen ce
India	Seven districts representing four agroclimatic zones of Maharashtra State	Apparently healthy native cattle, crossbred cattle and buffaloes; whole-blood samples	Molecular detection in vertebrate hosts	Species-specific and semi-nested PCR, with sequencing of representative amplicons	1,052 bovines; 92/1,052 positive for *T. orientalis* (8.7%). Regional prevalence ranged from 1.9% to 11.8%	Not determined	Subclinical carrier infection was common. Infection with tick-borne apicomplexans was associated with poorer body condition, particularly in crossbred cattle, although the effect could not be attributed solely to *T. orientalis*	Ticks were collected and identified, but competence for *T. orientalis* transmission was not experimentally assessed	[114]
	Three cattle farms in Karnataka State	Crossbred cows and indigenous bulls from lethal disease outbreaks	Molecularly confirmed clinical outbreaks with genotype characterisati on	Blood-smear examination; PCR and sequencing of MPSP, p23, 18S rRNA and ITS regions	Three lethal outbreaks; population prevalence was not assessed	Type 7 in crossbred cows and Chitose B, type 1, in indigenous bulls	Severe clinical disease and mortality, with reductions in haemoglobin and erythrocyte counts and biochemical abnormalities	Vector species and competence were not assessed	[99]

Country	Study area	Host or sample type	Evidence category	Diagnostic method	Sample size and prevalence	Genotype(s)	Clinical status or impact	Vector evidence	Reference
Iran	Guilan and Mazandaran Provinces, northern Iran	Indigenous cattle; whole-blood samples	Molecular detection and MPSP genotyping in vertebrate hosts	Nested PCR, amplification of the 18S rRNA and MPSP genes, sequencing and phylogenetic analysis	92 cattle sampled; a *T. orientalis* isolate was molecularly identified and genotyped; species-specific prevalence was not reported clearly	Buffeli	The parasite was detected during a survey of indigenous cattle; clinical effects attributable to the infection were not reported	Ticks were collected during the broader investigation, but transmission and vector competence were not demonstrated	[120]
	Four cities in West Azerbaijan Province	Apparently healthy cattle and water buffaloes; whole-blood samples	Molecular detection in vertebrate hosts	Giemsa-stained blood-smear examination and semi-nested PCR targeting the 18S rRNA gene	A *T. orientalis* isolate was molecularly identified and assigned to the Buffeli genotype; the study did not provide a clearly extractable species-specific prevalence	Not determined	Animals were native and apparently healthy; clinical impact was not assessed	Vector evidence was not assessed	[50]

Country	Study area	Host or sample type	Evidence category	Diagnostic method	Sample size and prevalence	Genotype(s)	Clinical status or impact	Vector evidence	Reference
Iraq	Fallujah City, Al-Anbar Governorate, western Iraq	Local-breed cattle; whole-blood samples	Molecular detection and sequence identification in vertebrate hosts	Giemsa-stained blood-smear examination, conventional PCR targeting the 18S rRNA gene, sequencing and phylogenetic analysis	100 cattle; 24% positive by microscopy and 73% positive for *Theileria* spp. by PCR. *T. orientalis* was identified among the sequenced isolates, but species-specific prevalence was not determined	Genotype not determined; 18S rRNA sequences assigned to *T. orientalis*	Sampled cattle showed signs compatible with theileriosis, including fever, icterus, lymph-node enlargement and lethargy; causation by *T. orientalis* specifically could not be established because several *Theileria* species were detected	Tick infestation was reported, but the responsible vector and its competence were not established	[115]
Nepal	Kathmandu Valley: Kathmandu, Lalitpur and Bhaktapur districts	Clinically ill cattle presented to a veterinary hospital; whole-blood samples	Molecular detection in vertebrate hosts	Giemsa-stained blood-smear examination, nested PCR targeting the 18S rRNA gene, sequencing and phylogenetic analysis	106 cattle; piroplasms detected in 45/106 by microscopy and 56/106 by nested PCR. *T. orientalis* was confirmed among sequenced samples, but a species-specific prevalence could not be calculated	Not determined	Sampled cattle presented fever, reduced appetite and other clinical signs; piroplasm-positive cattle had lower haemoglobin and erythrocyte counts, but effects could not be attributed specifically to *T. orientalis*	Vector competence was not assessed	[121]

Country	Study area	Host or sample type	Evidence category	Diagnostic method	Sample size and prevalence	Genotype(s)	Clinical status or impact	Vector evidence	Reference
Pakistan	Four districts of Punjab Province	Imported cattle from Australia, native Pakistani cattle and water buffaloes; whole-blood samples	Molecular detection and MPSP genotyping in imported and native vertebrate hosts	Conventional MPSP PCR, multiplexed tandem PCR and sequencing	246 animals: 147 imported cattle, 50 native cattle and 49 buffaloes. Multiplex PCR detected infection in 36/147 imported cattle (24.5%), 3/50 native cattle (6.0%) and 3/49 buffaloes (6.1%)	Not determined in the cattle survey	Clinical impact was not systematically evaluated; higher loads of pathogenic genotypes occurred mainly in imported cattle	Vector species and competence were not investigated	[122]
Sri Lanka	Nuwara Eliya, Polonnaruwa, Ampara and Jaffna districts	Cattle; whole-blood samples	Molecular detection in vertebrate hosts	Species-specific PCR followed by sequencing of representative amplicons	316 cattle; 169/316 positive for *T. orientalis* (53.5%). District-level prevalence ranged from 31.0% to 98.5%	Not determined in the cattle survey	Epidemiological survey; animals were not selected as clinical cases and specific productive effects were not assessed	Vector competence was not investigated	[124]
China	Zhangjiakou, Hebei Province	Confined dairy cattle, including clinically affected, recovered and asymptomatic animals	Molecularly confirmed clinical outbreak and experimental isolation from vertebrate hosts	Blood-smear examination; PCR targeting 18S rRNA and MPSP genes; sequencing; experimental inoculation	63 cattle sampled; 42/63 positive (66.7%)	Chitose and Ikeda; Ikeda was successfully isolated experimentally and considered the predominant pathogenic genotype	Severe piroplasmosis with anaemia and deaths was reported in confined dairy cattle	Tick vector and vector competence were not investigated in this outbreak	[21]

Country	Study area	Host or sample type	Evidence category	Diagnostic method	Sample size and prevalence	Genotype(s)	Clinical status or impact	Vector evidence	Referen ce
Japan	Aso and Kuma districts, Kumamoto Prefecture; Ishigaki, Iriomote and Yonaguni Islands, Okinawa Prefecture	Grazing Holstein and Japanese Black cattle	Molecular detection and MPSP genotyping in vertebrate hosts	Microscopy, haematocrit, ELISA, MPSP- and p23-targeted PCR, sequencing and genotype-specific PCR	70 cattle; 68/70 positive by MPSP-PCR (97.1%). Farm-level prevalence ranged from 94.3% to 100%	MPSP Chitose, Ikeda, Buffeli, types 4 and 5 were identified by sequencing; genotype-specific PCR demonstrated the circulation of five district types	Only one animal was anaemic; most positive cattle appeared to represent subclinical or carrier infections	Vector competence was not assessed in this study	[97]
Mongolia	Selected provinces and districts across Mongolia	Cattle blood and engorged ticks collected from cattle	Molecular detection and MPSP genotyping in vertebrate hosts and ticks	MPSP-targeted PCR, sequencing and phylogenetic analysis	District-level prevalence ranged from 8.8% to 66.6% in cattle and from 3.7% to 73.3% in ticks	Buffeli, Chitose, types 5, 7 and N3; types 3 and N3 were the most widely distributed	Clinical status and productive impact were not evaluated	Parasite DNA was detected in engorged ticks, but this does not independently demonstrate vector competence	[154]
South Korea	Hoengseong, Jeongeup, Namwon and Jeju Island	Apparently healthy cattle; whole-blood samples	Molecular detection and MPSP genotyping in vertebrate hosts	PCR targeting the MPSP gene, sequencing and phylogenetic analysis	138 cattle; 57/138 positive (41.3%)	Buffeli, Ikeda, Chitose and type 7	Sampled cattle were asymptomatic; clinical and productive effects were not assessed	Vector species and competence were not investigated	[155]

Country	Study area	Host or sample type	Evidence category	Diagnostic method	Sample size and prevalence	Genotype(s)	Clinical status or impact	Vector evidence	Reference
Cambodia	Kompong Speu and Ratanakiri Provinces	Draught cattle; whole-blood samples	Molecular detection and historical MPSP allele characterisation in vertebrate hosts	MPSP gene-specific and allele-specific PCR	137 cattle; 69/137 positive (50.4%). Prevalence was 43/56 (76.8%) in Kompong Speu and 26/81 (32.1%) in Ratanakiri	Historical MPSP allelic types: Thai type detected in 64/69 positive cattle, C type in 36/69 and B/Warwick type in 11/69; mixed infections were common; I/Ikeda type was not detected. These allelic categories are not directly equivalent to the current numbered MPSP genotypes	Clinical status and productive impact were not assessed	Vector species and competence were not investigated	[109]
Indonesia	West Java	Cattle-derived *Theileria* isolate	Molecular characterisation of an isolate from a vertebrate host	PCR amplification and sequencing of the p33 piroplasm surface antigen gene	Molecular characterisation of a selected isolate; population prevalence was not assessed	*T. orientalis/sergenti/buffeli*-related isolate; no modern MPSP genotype assignment	Clinical status and productive impact were not reported	Vector evidence was not assessed	[110]

Country	Study area	Host or sample type	Evidence category	Diagnostic method	Sample size and prevalence	Genotype(s)	Clinical status or impact	Vector evidence	Reference
Malaysia	All states of Peninsular Malaysia; 43 cattle farms	Cattle; whole-blood samples	Molecular detection in vertebrate hosts	PCR targeting the MPSP gene, sequencing of representative amplicons and phylogenetic analysis	1,045 cattle; 520/1,045 positive (49.76%)	MPSP sequences confirmed *T. orientalis*; genotype distribution was not clearly reported in the abstract	Epidemiological survey; specific clinical and productive effects were not assessed	Vector species and competence were not investigated	[111]
Myanmar	Five cities representing different geographical regions of Myanmar	Cattle; whole-blood samples	Molecular detection and genetic characterisation in vertebrate hosts	PCR targeting the MPSP gene, sequencing and phylogenetic analysis	713 cattle; 258/713 positive (36.2%)	Multiple MPSP genotypes were identified (Buffeli, Chitose, types 4, 5 and 7), demonstrating considerable genetic diversity	Clinical status and productive impact were not assessed	Vector species and competence were not investigated	[112]
Philippines	Laguna, Batangas, Rizal and Quezon Provinces, Southern Luzon	Cattle and water buffaloes; whole-blood samples	Molecular detection and phylogenetic characterisation in vertebrate hosts	Nested PCR targeting the 18S rRNA gene, followed by species-specific PCR, sequencing and phylogenetic analysis	412 cattle and 108 water buffaloes. *T. orientalis* was detected in 72/412 cattle (17.5%), including mixed infections; no *T. orientalis* was detected in buffaloes	MPSP type 1 (Chitose-related) and type 2 (Ikeda-related); one isolate clustered with a previously reported Philippine *Theileria* sp. sequence and remained unassigned to a recognised MPSP genotype	Clinical effects attributable specifically to *T. orientalis* were not assessed	*Rhipicephalus microplus* was observed in the study area, but its competence for transmitting *T. orientalis* was not demonstrated	[113]

Country	Study area	Host or sample type	Evidence category	Diagnostic method	Sample size and prevalence	Genotype(s)	Clinical status or impact	Vector evidence	Reference
Thailand	Chiang Rai, Phayao, Mae Hong Son, Khon Kaen, Maha Sarakham and Loei Provinces	Beef cattle; whole-blood samples	Molecular detection and MPSP genotyping in vertebrate hosts	Species-specific PCR, MPSP sequencing and phylogenetic analysis	329 cattle; 99/329 positive (30.1%)	Types 5 and 7	Clinical impact was not systematically evaluated; mixed infections with *Babesia* spp. were frequent	Lack of tick control was associated with infection, but the competent vector was not determined	[107]
Vietnam	Thua Thien Hue Province	Vietnamese yellow cattle, water buffaloes, sheep, goats and blood-sucking ticks collected from cattle	Molecular detection and MPSP genotyping in vertebrate hosts and ticks	MPSP-targeted PCR, sequencing and phylogenetic analysis	13/94 cattle (13.8%), 11/43 buffaloes (25.6%), 1/21 sheep (4.8%) and 9/85 ticks (10.6%) were positive; goats were negative	Buffeli, Chitose, Types 5, 7, N1, N2 and N3	Clinical status and productive impact were not evaluated	Parasite DNA was detected in ticks, but detection alone does not establish vector competence	[108]
Australia	Nineteen cattle farms in Victoria	Symptomatic and asymptomatic cattle following oriental theileriosis outbreaks	Molecularly confirmed clinical outbreaks and epidemiological investigation	Blood-smear microscopy; PCR targeting the MPSP gene; mutation scanning, sequencing and genotype characterisation	301 cattle; 213/301 positive by PCR (70.8%) and 79/281 positive by microscopy (28.1%)	Ikeda in 91.1%, Chitose in 32.9%, Buffeli in 2.4% and type 5 in 1.4% of PCR-positive cattle; Ikeda–Chitose mixed infections occurred in 21.6%	Disease mainly affected beef cows older than two years during the pre-calving period and was associated with abortion and cow mortality	Vector competence was not directly investigated in this study	[102]

Country	Study area	Host or sample type	Evidence category	Diagnostic method	Sample size and prevalence	Genotype(s)	Clinical status or impact	Vector evidence	Reference
	Ten dairy farms in south-western Western Australia	Apparently healthy lactating dairy cows	Molecular detection and genotype-specific surveillance in vertebrate hosts	Genotype-specific quantitative PCR	100 cows; 13/100 positive (13%); positive cattle were detected on 6/10 farms	Ikeda was detected in 11 cows and Buffeli in one; one positive sample could not be genotyped conclusively	Cows were apparently healthy; clinical and productive effects were not assessed	Tick vector and vector competence were not investigated	[104]
New Zealand	Three pasture-based spring-calving dairy farms in the North Island	Dairy cows monitored longitudinally during the 2014–2015 milking season	Longitudinal molecular detection and assessment of productive and reproductive impact in vertebrate hosts	Ikeda-specific quantitative PCR, haematocrit measurement and longitudinal production and reproduction records	Initial prevalence was 100% on Farm A, 57% on Farm B and 26% on Farm C	Ikeda	Chronic carrier infection was not associated with significant milk or reproductive losses, but incident infections were associated with a reduction of approximately 266 kg of energy-corrected milk	Vector competence was not experimentally assessed within this study	[103]
